# Modular nest structure influences activity cycle synchronisation in ant colonies

**DOI:** 10.1038/s42003-026-10560-x

**Published:** 2026-06-27

**Authors:** Thomas O. Richardson, Matthias Rüegg, Zoe Pritchard, Laurent Keller, Nathalie Stroeymeyt

**Affiliations:** 1 School of Biological Sciences, University of Bristol, Bristol, UK; 2 Department of Ecology and Evolution, University of Lausanne, Lausanne, Switzerland; 3 Institute of Mechanical Engineering, EPFL, Lausanne, Switzerland; 4 Bozeman Field School, Bozeman, MT USA; 5 Department of Ecology, Evolution and Organismal Biology, Iowa State University, Ames, IA USA; 6 Social Evolution Unit, Chesières, Switzerland

**Keywords:** Behavioural ecology, Oscillators

## Abstract

Periodic oscillations are widespread in biological systems, from heartbeats to predator-prey cycles. However, although oscillatory systems often exhibit modular organisation, how cycles are generated and coordinated across modules remains unclear. Here, we tested how modular nest architecture affects short-term activity cycles in the acorn ant *Leptothorax acervorum*. Activity cycles were consistently initiated by spontaneous pacemakers in the inner parts of the nest and transmitted toward the outer regions by intermediate ‘transfer’ ants. Compared to single-chamber nests, modular (double-chamber) nests induced partial decoupling of activity between chambers, resulting in reduced colony-wide rhythmicity. However, increased movement speeds of transfer ants in double-chamber nests relative to single-chamber nests enhanced activity transmission, partly restoring coordination between chambers. As well as illustrating how modular structure shapes collective dynamics, our results shed light on how ant colonies solve a fundamental challenge facing many biological systems: maintaining coordination between physically-segregated units.

## Abstract (web summary)

Automated tracking shows that modular nest architecture partially decouples activity cycles in ants, but transfer ants restore coordination between chambers, revealing how biological systems can maintain synchrony across physically-segregated units.

## Introduction

Modular organisation—the subdivision of complex systems into distinct yet interconnected functional units—is pervasive across biological scales, from compartmentalised metabolic networks to ecosystems composed of loosely connected groups of closely interacting species^1^. Modularity is thought to have facilitated the evolution of biological complexity, as compartmentation and parallelisation improve efficiency within modules while increasing system-wide resilience, which in turn promotes the emergence and diversification of new modules with novel functions. Consequently, modular organisation is considered a key driver of the Major Evolutionary Transitions^1–3^. However, in self-organised systems without centralised control, modularity may hinder communication, coordination, and resource flow between units. Such systems often depend on compensatory mechanisms to maintain cross-module coupling^4–6^. This challenge is particularly significant in rhythmically oscillating systems that require system-wide synchronisation, such as the circadian clock or oscillatory activity in the brain^7,8^.

Cyclical patterns of activity occur at all levels of biological organisation, from intracellular calcium oscillations that coordinate signalling processes^9^, rhythmic cardiac and respiratory activity in vertebrates^10^, and circadian rhythms that regulate daily activity patterns in organisms^8^, to regular fluctuations in prey and predator populations within ecosystems^11,12^. Notably, many rhythmical biological systems are compartmentalised into functional modules, which entrain one another to maintain synchrony, and the mechanisms underlying the initiation and cross-module coupling of activity have been particularly well studied in mammalian physiology. In the heart, for instance, the sinoatrial node acts as the primary pacemaker, entraining other components such as the atrioventricular node and Purkinje fibres, even though these can also generate spontaneous rhythms when isolated^13–15^. Similarly, the suprachiasmatic nucleus (SCN) in the hypothalamus functions as the master circadian pacemaker, coordinating semi-autonomous peripheral oscillators throughout the body to produce a single homogeneous body clock^8,16^. In the brain, distinct cortical and subcortical regions exhibit independent oscillatory activity but synchronise during complex cognitive tasks^4,7^. By contrast, little is known about the coordination of activity rhythms at higher levels of biological organisation – for example, within societies that are structured into communities, each with distinct activity patterns^12^. Here, we address this gap by investigating how modular organisation influences the generation and synchronisation of collective activity cycles in acorn ant (*Leptothorax acervorum*) colonies, using experimental manipulations of nest architecture.

Colonies of *Leptothorax*, *Temnothorax*, and *Tapinoma* ants exhibit self-organised, quasi-periodic oscillations in collective activity, typically displaying three to four activity peaks per hour^17–25^. Several hypotheses have been proposed to explain the function of these cycles. They may enhance ergonomic efficiency in tasks such as brood tending^26,27^, allow respiratory carbon dioxide to dissipate during periods of inactivity^28^, or help eliminate outdated information^22^. Alternatively, these cycles might be non-adaptive epiphenomena, that is, emergent by-products of other colony-level processes^19^. In addition to heterogeneous temporal activity patterns, many ant species also exhibit heterogeneous spatial organisation, building nests that are divided into multiple chambers^29–41^. Even in species that inhabit pre-existing cavities rather than constructing elaborate nests, spatial complexity can still arise. Colonies of *Leptothorax* ants typically inhabit rock crevices, decaying wood, or acorns^42,43^. Although these nest sites are often described as single internal spaces, structural analyses (e.g., CAT scans of acorn nests) reveal substantial internal complexity, including folds and invaginations that create partial spatial subdivision^44^. Colonies nesting in decaying wood also often occupy multiple cavities connected by a network of galleries. These features generate a degree of spatial modularity even in nests formed from pre-existing cavities. Such modular architecture has important functional consequences for ant colonies: limiting pathogen spread^45–47^, regulating the flow of information and resources^39^, and providing dedicated zones for specific tasks^48,49^. However, the potential effects of nest modularity on collective activity dynamics remain unexplored. Here, we investigated how modular spatial organisation influences rhythmic temporal organisation in *L. acervorum* colonies. Using barcode-based automated tracking^47,49–51^ and architectural nest manipulations, we aimed to: (i) investigate how modular nest architecture affects social, spatial, and temporal organisation; (ii) identify the individuals responsible for initiating and propagating collective activity; and (iii) uncover potential compensatory mechanisms that maintain coordination between spatially segregated nest compartments.

## Results

**Fig. 1:**
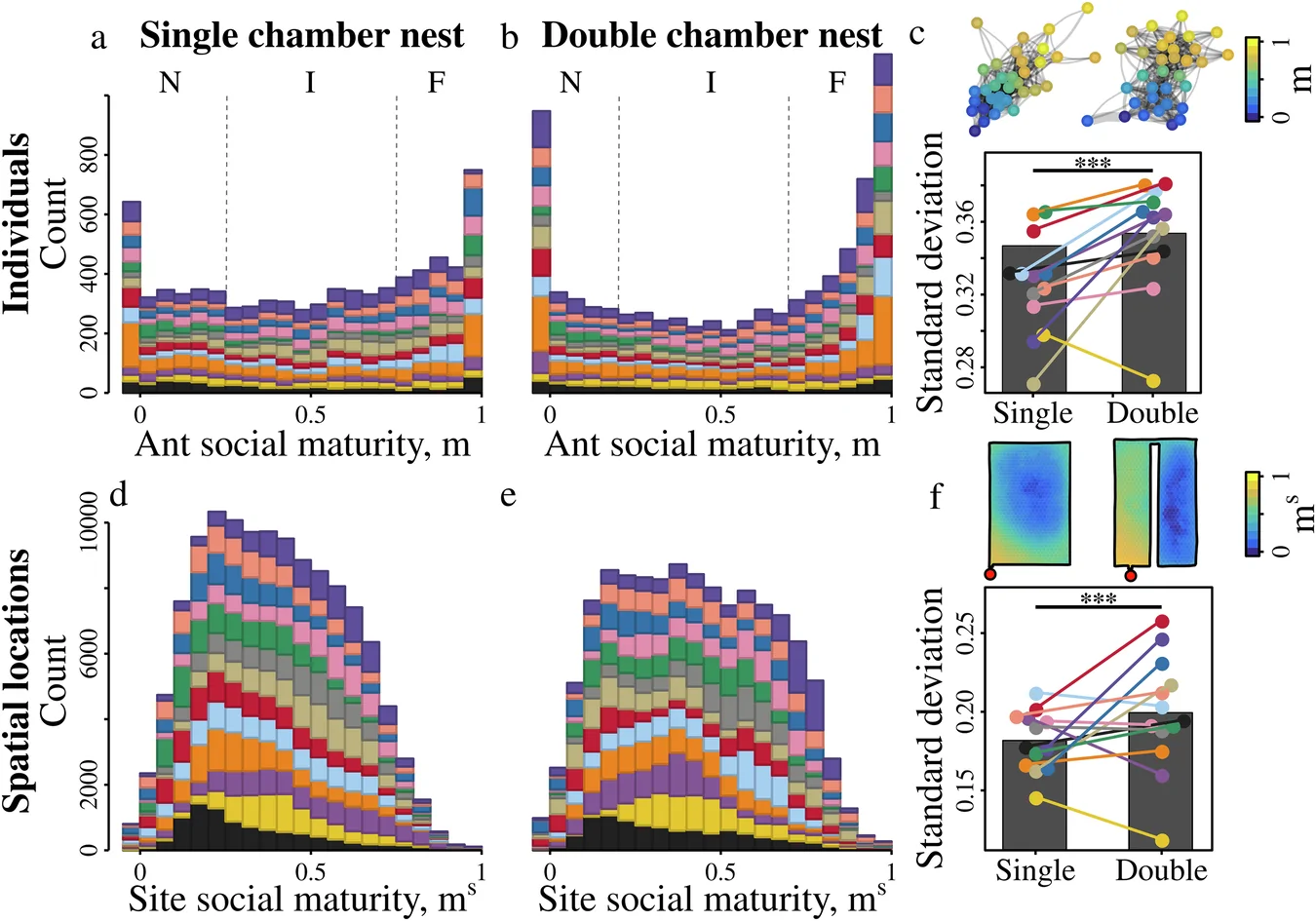
Nest architecture influences social and spatial organisation. Stacked barplots show the distributions of social maturity for individual ants (**a**, **b**) and for the typical visitor to each spatial location (**d**, **e**), for colonies in single and double-chamber nests. Colours indicate colony identities. Barplots show the standard deviations of the social maturity distributions, for individuals (**c**) and for spatial locations (**f**). Each coloured point shows the standard deviation of the colony-level social maturity distribution. Bars represent the grand mean, calculated across all colonies. ***: *p* < 0.0001 (LMM). *n* = 13 independent colonies, each tested under both nest architectures in a paired crossover design. Statistical models were fitted to social- and site-maturity distribution heterogeneity calculated separately within each 12-hour recording session (*n* = 311). Dashed vertical lines indicate the thresholds used to distinguish Nurses (N), Intermediates (I) and Foragers (F). The networks above panel (**c**) show the contact networks for one colony in both nest architectures, calculated across all 12-hour recording sessions. Nodes represent individual ants, and are coloured according to the social maturity, *m*. Edges represent the number of contacts between a given pair. The network layout is determined by a force-directed algorithm which ensures that pairs that have many contacts are neighbours. The nest maps above (**f**) show the site maturities, *m*^*s*^, for one colony in the single-chamber nest (left) and the double-chamber nest (right). The red dots indicate the entrance tunnel.

To study the effect of modular nest architecture on colony organisation and synchronisation, we conducted a barcode-based automated tracking experiment using 13 whole colonies of *L. acervorum* ants (Table S1). We used a paired crossover design in which each colony was successively housed in two different nest architectures of equal total area: a single-chamber nest consisting of one large chamber, and a double-chamber nest consisting of an inner and an outer chamber connected by a passage, with each chamber having half the area of the single-chamber nest (Fig. 1 and Fig. S1; Methods). To balance potential order effects, seven colonies started in the single-chamber nest and six in the double-chamber nest. All colonies were tracked continuously for six days in the first architecture, after which they were transferred to the alternative architecture and tracked for an additional six days. Experiments were conducted under 12:12 h cycles of light and temperature (25 °C day / 20 °C night). For the analyses described below, the continuous recordings were divided into successive 12-hour daytime (07:30-19:30) and nighttime (19:30-07:30) periods, resulting in twelve 12-hour recording sessions per architecture (Fig. S1). The nests were covered by an infra-red-pass filter, allowing tracking under infra-red illumination while keeping the nest interior dark to the ants (see Methods). Altogether, the experiment produced 152 days of continuous tracking, representing over 4000 ant-days and 7 × 10^8^ individual trajectory coordinates (Table S1).

### Modular nest architecture promotes social and spatial differentiation

We first quantified the effect of modular nest architecture on the social and spatial organisation of the colony. To quantify social organisation, we extracted physical contacts between all pairs of individuals using an established geometrical approach^47,50,52^. Social networks were produced by aggregating all contacts within each 12-hour recording session, resulting in twelve networks per colony per nest architecture (Fig. S2-S3). Applying a soft community detection algorithm (Facetnet^53^) to each network revealed that all colonies were structured into two overlapping communities (Fig. 1 and Fig. S4): a nurse community (N) centred on the brood pile and a forager community (F) occupying the area around the nest entrance. As social insect workers typically transition from inside- to outside-nest tasks (e.g., brood tending and foraging respectively) as they age^48,49,54^, we used the Facetnet score for the forager community as a measure of the *social maturity* of each ant^55–57^ (see Methods).

In both nest architectures, worker social maturity followed a qualitatively similar bimodal distribution, with one peak for full nurses, one peak for full foragers, and an intervening trough representing generalists affiliated to both groups (henceforth *intermediates*; Fig. 1a, b). However, the left and right peaks appeared more prominent in the double-chamber nests, suggesting that modular nest architecture induces a stronger polarisation of workers towards the full nurse and forager profiles. To test this hypothesis, we quantified the heterogeneity of each distribution using the standard deviation and seven other inequality metrics (Fig. 1c and Fig. S5). We found that the social maturity distributions of colonies housed in double-chamber nests were significantly more heterogeneous than those in single-chamber nests for seven out of eight metrics (general linear mixed-effect model (LMM) with colony and session number as random factors; effect of architecture on standard deviation, d.f.=1, *χ*^2^=78.7, *p* < 0.0001; see Fig. S5 for other metrics). This suggests that modular nest organisation is associated with greater social differentiation among workers, which is consistent with the prediction that modularity should promote functional diversification^1,2^.

To quantify spatial organisation, we divided the nest into a regular hexagonal grid (grid width = 1/2 worker body length), counted the visits of each worker to each grid site, and used the visitors’ social maturity scores to calculate the social maturity of the typical visitor to each site (*site maturity*; Methods, Figs. S6-S7). Site maturity ranged from 0 (site solely visited by full nurses) to 1 (site solely visited by full foragers). Unlike the individual social maturity distributions, the site maturity distributions were unimodal (Fig. 1d, e), with few exclusive sites visited mainly by specialists of a single task group, and many communal sites visited by a mixture of ants (1/4≤ site maturity ≤3/4). Comparing the site maturity distributions between nest architectures revealed that colonies in double-chamber nests exhibited greater spatial heterogeneity than those in single-chamber nests for five out of eight heterogeneity metrics (Fig. 1f; LMM, effect of architecture on standard deviation, d.f.=1, *χ*^2^=19.8, *p* < 0.0001; see Fig. S5 for other metrics). Furthermore, double-chamber nests contained significantly fewer communal sites (LMM, d.f.=1, *χ*^2^=7.97, *p* = 0.0048). Together, these analyses indicate that modular nest organisation also increases spatial segregation between task groups by shrinking the communal areas and enlarging exclusive areas.

**Fig. 2:**
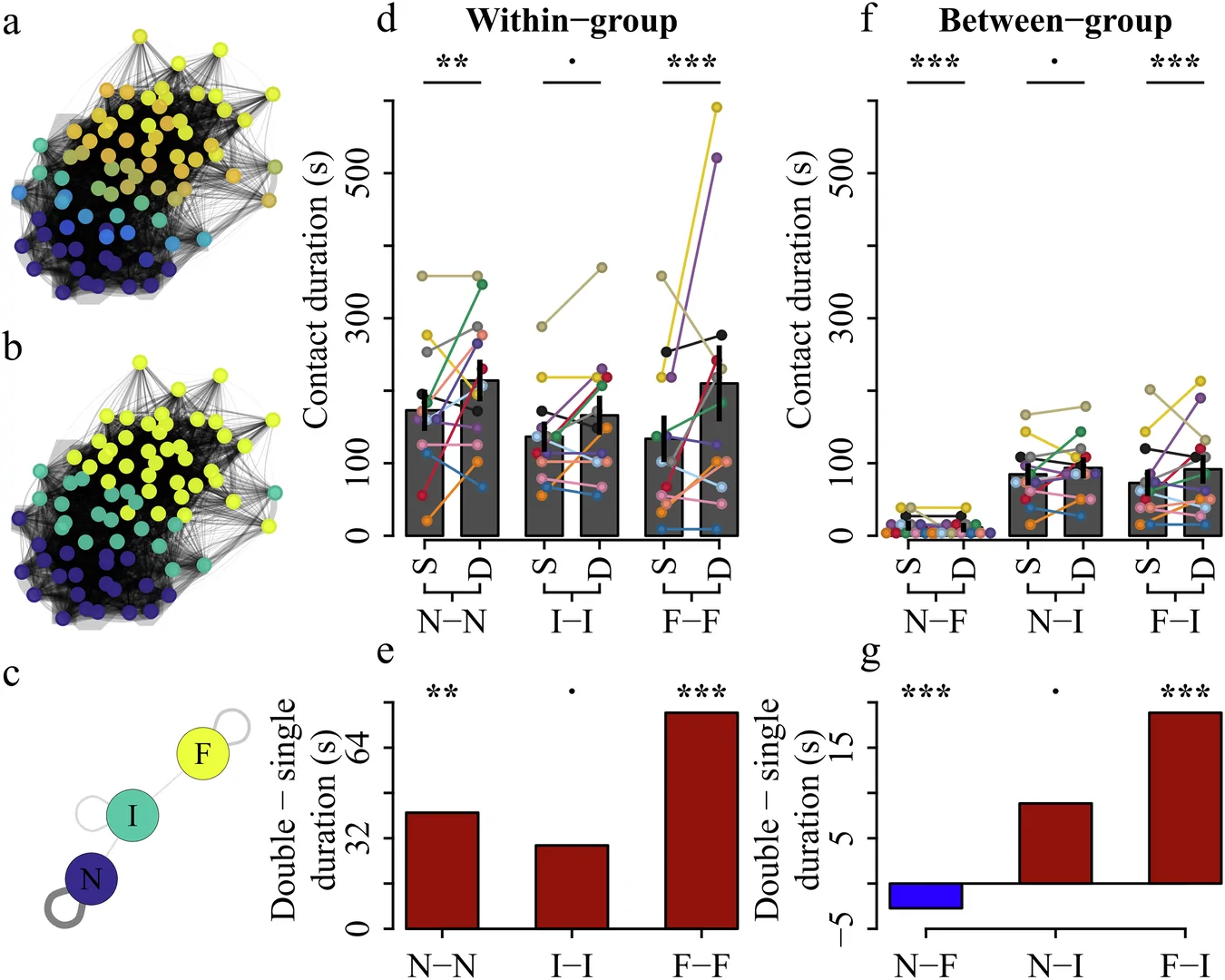
Modular nest architecture modifies within-group and between-group connectivity. **a–c** Procedure for measuring the connection strengths within and between task groups. **a** An example 12-hour contact network (Colony 29, double-chamber nest, day 8). Node colours indicate the social maturity, *m*. Edge shading is proportional to the total duration of contact between each pair of ants. **b** The 12-hour contact network with discretised task labels. Nurse specialists (those with *m* < 1/4) were assigned a `N' label (blue), forager specialists (those with *m* > 3/4) were assigned a `F' label (yellow), and all other workers were assigned an `I' (Intermediate) label (green). **c** Coarse-grained representation of the contact network. Curved edges represent within-group connection strengths, while straight edges represent between-group connection strengths. Edge shading is proportional to the median contact duration across all pairs. **d**, **f** Median contact duration across all within-group (**d**) or between-group (**f**) ant pairs, conditioned on the nest architecture (S: single-chamber, D: double-chamber). Points show colony means. Bars and whiskers show grand means and standard errors. *n* = 13 independent colonies, each tested under both nest architectures in a paired crossover design. Statistical models were fitted to median total contact durations across ant pairs, calculated separately for each task-group pairing within each 12-hour contact network (*n* = 311). Asterisks indicate Benjamini-Hochberg corrected post-hoc contrasts for the model described in the text (.: *p* < 0.1; **: *p* ≤ 0.001; ***: *p* ≤ 0.0001). **e**, **g** Between-architecture differences in within-group (**e**) and between-group contact duration (**g**). Bars show the mean pairwise differences between the data points shown in (**d**, **f**) (*n* = 13 independent colonies). Asterisks as in (**d, f**).

Next, we tested the effect of nest architecture on within- and between-community connectivity. To do so, we counted the number of contacts and the total contact duration for each ant pair in each 12-hour contact network, and then used the community affiliations of the pair members to classify the pairs into categories (*within* task groups, nurses: N-N; intermediates: I-I; foragers: F-F; *between* groups: N-F, N-I, F-I; Fig. 2 and Fig. S8). We found that connectivity within task groups was stronger in double-chamber than in single-chamber nests, especially within the two specialised task groups (nurses and foragers; Fig. 2d-e, LMM, log_10_duration  ~ architecture  × edge type, within-group contacts, interaction edge: architecture, d.f.=2, *χ*^2^=11.36, p = 0.0034; post-hoc contrasts with Benjamini-Hochberg correction, double-chamber minus single-chamber, N-N: z=3.94, p = 0.00012; I-I: z=1.92, p = 0.055; F-F: z=6.66, p < 0.0001). This is consistent with predictions that modular organisation should foster integration and coherence within modules^1^. By contrast, connectivity between nurses and foragers was significantly lower in double-chamber than in single-chamber nests (contact duration: Fig. 2f-g, LMM, log_10_duration  ~ architecture  × edge type, between-group contacts, interaction edge: architecture, d.f.=2, *χ*^2^=45.23, *p* < 0.0001; post-hoc contrasts with Benjamini-Hochberg correction, double-chamber minus single-chamber, N-F: z=-5.07, *p* < 0.0001), in agreement with the expectation that modular organisation should inhibit information flow between specialised units^1,2^. However, interestingly, compared to their behaviour in single-chamber nests, intermediate workers in double-chamber nests increased their contacts with both foragers (F-I: z=4.07, *p* < 0.0001) and—to a lesser extent—nurses (N-I: z=1.74, *p* = 0.082; Fig. 2f, g). All trends were qualitatively similar when considering number of contacts rather than duration (Fig. S8). This suggests that the intermediate workers may play an important role as *transfer workers*^58^ that maintain information flow between spatially- and socially-segregated communities in modular nest architectures.

### Modular nest architecture weakens spatial activity coupling

We next explored how the characteristic short-term activity cycles produced by *L. acervorum* are influenced by modular nest architecture, and quantified the degree of activity coupling between different parts of the nest. To characterise collective activity, we classified each ant as active or inactive at any given time using the individual-level trajectories (Methods; Fig. S9), and constructed time series of the proportion of active ants within a given area of interest (e.g., the whole nest, or a chamber). We then used spectral analysis to identify the typical time between cycle peaks (i.e., the dominant period, *λ*), and to measure the associated rhythmicity (i.e., the amplitude of the dominant period, *β*^25^).

**Fig. 3:**
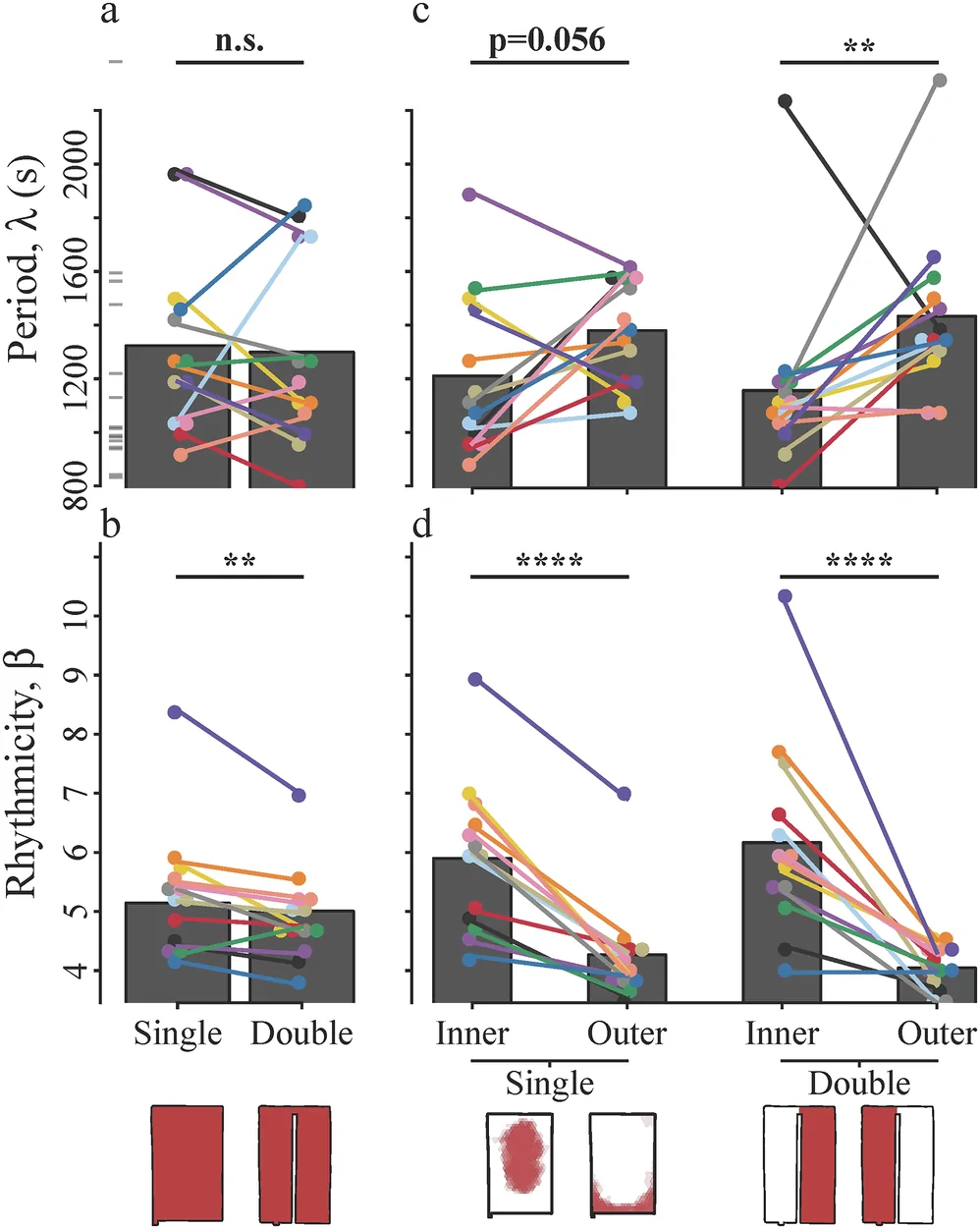
Modular nest architecture allows activity decoupling within the nest. Period (**a**) and rhythmicity (**b**) of whole-nest activity cycles for colonies in single or double-chamber nests. The grey horizontal lines in (**a**) indicate the activity cycle periods of 15 *L. acervorum* colonies in single-chamber nests, from ref. ^22^. **c**, **d** Period and rhythmicity of activity cycles within the inner versus the outer spatial zone of colonies in single-chamber nests, and the inner versus the outer chambers of those in double-chamber nests. The coloured nest layouts below (**d**) depict the relevant location; inner/outer spatial zones for single-chamber nests, and inner/outer chambers in double-chamber nests. In all panels, coloured points indicate colony means, obtained by averaging across all 12-hour sessions. *n* = 13 independent colonies, each tested under both nest architectures in a paired crossover design. Statistical models in (**a**, **b**) were fitted to one period or rhythmicity value per 12-hour whole-nest activity time-series (*n* = 311 sessions). Statistical models in (**c**, **d**) were fitted to paired inner- and outer-location values calculated from each 12-hour activity time-series (*n* = 311 sessions; 622 location-level observations). In all panels, symbols indicate statistical significance of main effects (a-b) or Benjamini-Hochberg corrected post-hoc contrasts (**c**, **d**). **n.s**.: *p* > 0.1; **: *p* < 0.01; ****: *p* < 0.0001.

Analyses of the whole-nest time series confirmed that colonies displayed the characteristic  ~ 20 min activity cycles previously reported in *Leptothorax* ants^17–19,21–24^ regardless of whether they were housed in single or double-chamber nests (Fig. 3a). However, although nest architecture did not affect the dominant period of the whole-nest time-series (Fig. 3a; LMM, *λ* ~ architecture; *F*_1,286_=0.009, *p* = 0.92), it did affect the rhythmicity, with colonies in double-chamber nests exhibiting less prominent cycles than those in single-chamber nests (Fig. 3b; LMM, *β* ~ architecture; *F*_1,287_=7.7, *p* = 0.0057).

As the whole-nest time-series could potentially mask differences in activity patterns between different locations, we next tested for spatial variation in activity patterns. To do so, for double-chamber nests we compared the activity time-series between the inner and outer chambers. For single-chamber nests, we used the site maturity scores to define two spatial zones of equal area: an inner zone with low site maturity, mainly occupied by nurses, and an outer zone with higher site maturity, mainly occupied by foragers (Methods; Fig. S6 and Fig. S10). This division closely replicated the socio-spatial organisation of double-chamber nests, where the inner chamber is mainly occupied by nurses and the outer chamber by foragers (Fig. S7 and Fig. S10), thus allowing for meaningful comparisons between architectures. In both nest architectures the activity cycles were faster in the inner location than in the outer location; however, these differences were only statistically significant in the double-chamber nest (Fig. 3c; LMM, *λ* ~ architecture  × location; single-chamber, model-estimated *λ*_out._ − *λ*_in._ = 169 ± 88 s; planned post-hoc contrasts with Benjamini-Hochberg correction: z=1.9, p=0.056; double-chamber, *λ*_out._ − *λ*_in._ = 275 ± 89 s; post-hoc contrast: z=3.1, *p*=0.0038). Furthermore, in both architectures, the cycles in the inner location were more prominent than those in the outer location (Fig. 3d; LMM, *β* ~ architecture  × location; single-chamber, *β*_out._ − *β*_in._ = -1.6 ± 0.2, post-hoc contrast; single-chamber, z=10.3, p < 0.0001; double-chamber, *β*_out._ − *β*_in._ = -2.1 ± 0.2, post-hoc contrast, z=13.3, p < 0.0001).

**Fig. 4:**
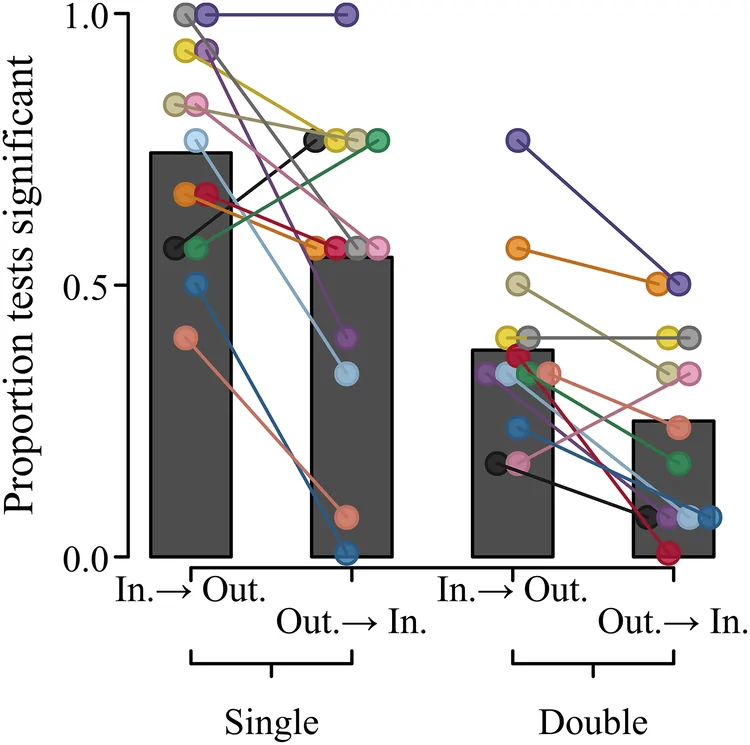
Modular nest architecture weakens within-nest activity coupling. The y-axis gives the proportion of 12-hour sessions for which either the inner activity Granger caused the outer activity (In. → Out.), or vice-versa (Out. → In.). Points show colony-level proportions of significant Granger causality tests. *n* = 13 independent colonies, each tested under both nest architectures in a paired crossover design. The binomial GLMM was fitted to binary Granger-test outcomes for each causal direction within each 12-hour session (*n* = 311 sessions; 622 direction-level test outcomes). All time-series were coarse-grained into 1-minute bins, and the history length (the lag) was set to 2 min. A parameter sweep across a range of time bins and lags confirmed that the direction of the differences between the inner and outer locations, and between the two directions, was largely insensitive to the precise parameter used (Fig. S11).

We next used Granger causality testing^59^ to formally quantify the degree of activity coupling between the inner and outer locations in both nest architectures. Granger causality evaluates the degree to which knowing the current value of a time series *X* improves predictions of the future values of another time series *Y*, and infers causality between the two (*X* ‘Granger-causes’ *Y*) based on the assumption that the cause precedes the effect. We used this approach to perform two hypothesis tests for each 12-hour session: one testing whether the activity in the inner location Granger-causes the activity in the outer location (In. → Out. direction), and another testing the reverse (Out. → In.). There were significantly more statistically significant Granger tests for the In. → Out. than for the Out. → In. direction in both nest architectures, indicating asymmetrical, directional coupling from the inner to the outer location (Fig. 4; binomial GLMM, binary-coded test result  ~ architecture × direction with colony, session number and day/night as random factors; effect of coupling direction, d.f.=1, *χ*^2^=19.54, *p* < 0.0001; interaction architecture:coupling direction, d.f.=1, *χ*^2^=0.59, *p* = 0.44). Furthermore, there were significantly more statistically significant Granger tests in single-chamber nests than in double-chamber nests, regardless of direction (effect of architecture, d.f.=1, *χ*^2^=71.36, *p* < 0.0001). This shows modular nest organisation weakens activity coupling between physically segregated ant populations.

### Activity pacemaking

We next investigated how activity cycles are generated and propagated between different parts of the nest. First, we examined whether workers’ transitions between inactive and active states—referred to here as activation and deactivation—were consistent with a contagious process, in which activity spreads through physical contacts with nestmates^60^. To do so, we used a multivariate approach^49^ to identify the times when each ant was active (see Methods), and then tested whether the probability of an ant activating during a colony-wide activity trough (*P*_*activation*_), or deactivating during a peak (*P*_*deactivation*_), was associated with the number of recent contacts with active or inactive individuals (Fig. S12).

We found a strong positive association between *P*_*activation*_ and the number of recent contacts with active nestmates, with each additional contact increasing the odds of activation by 2.5 times (Fig. S13, Table S2). This indicates that activation spreads via social contagion, whereby active ants bumping into inactive ants cause the latter to activate^60^. By contrast, *P*_*deactivation*_ showed no positive association either with the number of recent contacts with *active* nestmates or with the number of recent contacts with *inactive* nestmates (Fig. S13, Table S2). This suggests that deactivation does not spread socially via classical social contagion (i.e., positive feedback) or via reverse contagion^61^ (i.e., negative feedback). Hence, in the remainder of the analysis we focus on activation only.

As our earlier Granger causality analysis revealed asymmetrical activity coupling between the inner and outer locations (Fig. 4), we next examined whether tendencies to activate spontaneously (i.e., in the absence of a social trigger), versus via social contagion (i.e., through contact with an active nestmate) varied across space. To do so, we classified each activation as either *spontaneous* (no contact with active nestmates in the minute immediately preceding the activation) or *induced* (at least one contact). For each individual, we then calculated an index of spontaneity, *IoS*, defined as the proportion of spontaneous activations among all activations (see Methods).

We found that IoS was influenced by nest architecture, location, and their interaction (generalised linear mixed model, GLMM, with binomial error: *IoS*  ~ architecture  × location; effect of architecture, d.f.=1, *χ*^2^=1664, *p* < 0.0001; effect of location, d.f.=1, *χ*^2^=2572, *p* < 0.0001; architecture:location interaction, d.f.=1, *χ*^2^=139, *p* < 0.0001). Within both nest architectures, ants had a greater bias towards spontaneous activation (i.e., a greater *IoS*) in the inner than in the outer location, suggesting that the activity cycles originate there. Furthermore, this difference was more pronounced in the double-chamber nests (post-hoc contrasts with Benjamini-Hochberg correction, single-chamber nests, odds ratio for *IoS*_in._/*IoS*_out._=1.25 ± 0.01, z=28.3, *p* < 0.0001; double-chamber, *IoS*_in._/*IoS*_out._=1.41 ± 0.01, z=46.7, *p* < 0.0001).

**Fig. 5:**
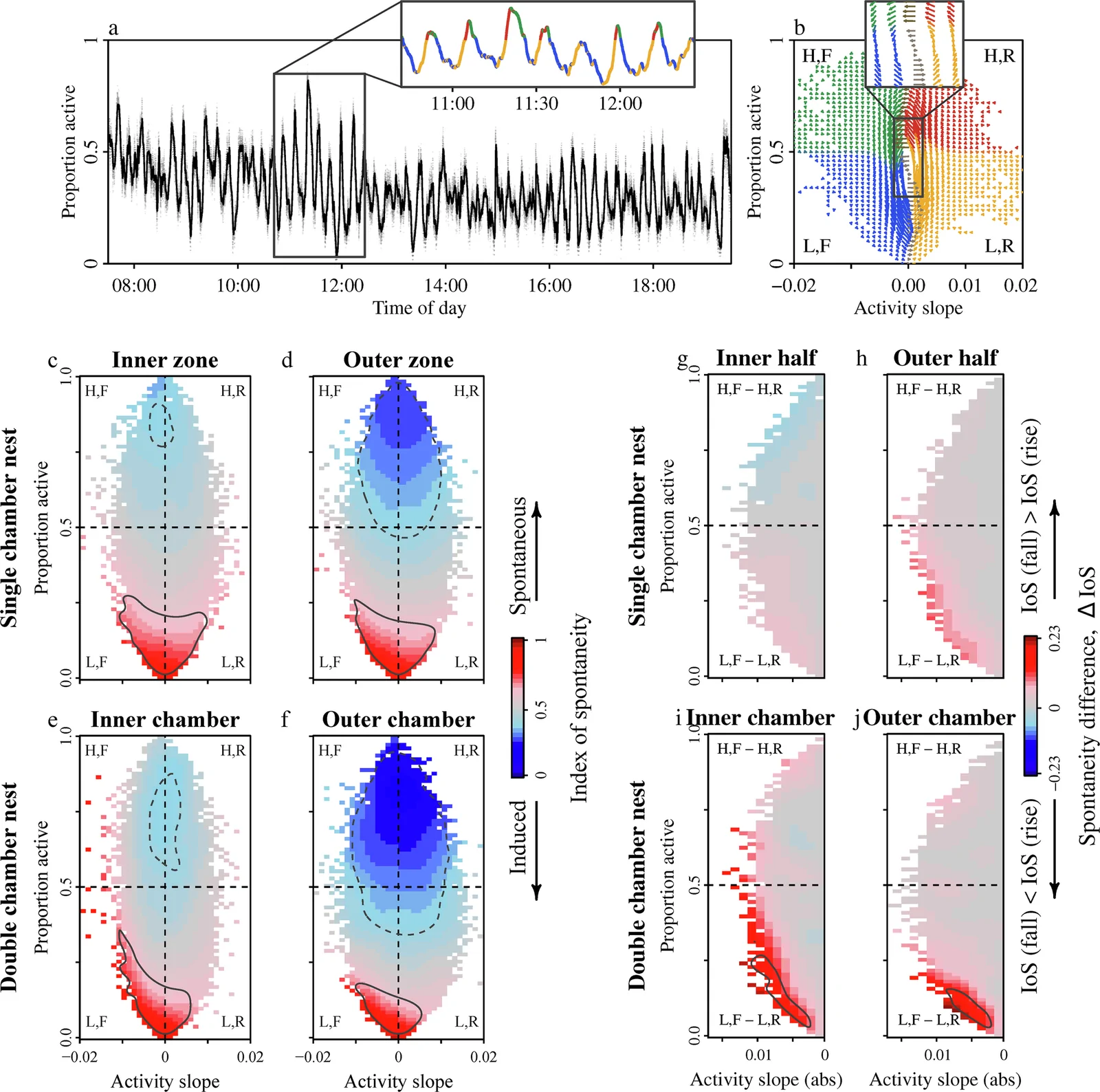
Mapping the distribution of spontaneous activation over the activity cycle suggests that inner-chamber ants act as pacemakers. **a** Example 12-hour whole-nest activity time-series (colony 29, single-chamber nest, day 8). Grey points: raw data. Solid black line: 60-second moving average. Colours indicate the activity level and the sign of the slope. Yellow: low and rising (L,R), red: high and rising (H,R), green: high and falling (H,F), blue: low and falling (L,F). **b** Phase-space representation of overall activity level and activity slope dynamics. Arrows represent polar vectors, for which the orientations indicate the typical change in activity between time *t* and *t* + 1. Arrow lengths are inversely proportional to the directional variance, with long arrows denoting locations with strong directional consistency. Vectors were calculated by averaging across the 156 whole-nest 12-hour time-series for colonies in the single-chamber nest. **c–f** Index of spontaneity as a function of the position in the phase-space. Cell colours indicate the estimates from the fitted GAMM models described in the text. The GAMM underlying (**c–j**) was fitted to binned ant-level off-to-on transition data, weighted by the number of activation events contributing to each activity-slope/activity-level bin. The displayed analysis used *n* = 13 independent colonies, 301 12-hour sessions, 769 ant identities, 713,490 binned model-input rows and 776,144 underlying off-to-on activation events. Contours indicate regions in which there is significant over- or under-representation of spontaneous activation (i.e., areas where the model-estimated 95% confidence intervals do not encompass *IoS* = 1/2). Dashed lines indicate the separation between the four activity-phase quadrants (horizontal line: proportion active = 0.5; vertical line: slope = 0; used for visual interpretation only). **g–j** Difference maps, produced by subtracting the half of the activity map representing rising activity (positive activity slopes, right side), from the half representing falling activity (negative slopes, left side). Positive values of Δ*IoS* indicate that falling activity is associated with greater spontaneous activation than rising activity. Contours indicate areas where the *IoS* for falling activity significantly differs from the *IoS* for rising activity.

As spontaneous activations might exert a greater pacemaking effect if they occur during activity troughs than during activity peaks, we next investigated how activation spontaneity varies across the activity cycle. To do so we constructed a semi-parametric model describing how IoS changed according to the colony’s position in the activity *phase space*, defined by the current activity level and its slope (Methods; Fig. 5a-b). To visualise how *IoS* varies according to the activity context, we used the fitted model to identify regions of the activity phase space with a significant bias towards spontaneous (*IoS* > 1/2) or towards induced activations (*IoS* < 1/2). In all four architecture-location combinations, spontaneous activations were significantly over-represented at low activity levels, while induced activations were significantly over-represented at high activity levels (Fig. 5c–f; solid and dashed contours, respectively).

Notably, the regions of elevated activation spontaneity (red regions in Fig. 5c–f) were skewed toward the lower-left quadrant of the phase space, that is, during periods of low and falling activity just before the start of a new cycle. To quantify the number of workers contributing to the initiation of each cycle, we counted the number of spontaneous activations occurring within the 60 s preceding each activity trough. Across the 26,910 detected troughs, this yielded a mean of 2.5  ± 0.2 ants per trough (mean  ± SE). The number of ants activating spontaneously increased significantly with colony size (GLMM with Poisson error; effect of colony size: d.f.=1, *χ*^2^= 175.67, *p* < 0.0001), ranging from 1.85 ants per trough in the smallest colonies (*N* = 33 workers) to 5.3 ants per trough in the largest colonies (*N* = 104 workers). Thus, the initiation of each cycle typically involved several workers rather than a single individual.

We next examined whether the *direction of change* in activity affects *IoS*, as this would indicate that the recent social context influences a worker’s decision to activate spontaneously, and consequently, its likelihood of initiating a new cycle. To do so, for each activity level we calculated the difference in *IoS* between periods when activity was falling (left-hand-side of the activity maps) versus rising at the same rate (right-hand-side), Δ *IoS*. In single-chamber nests, Δ*IoS* values were close to 0 throughout the phase space (Fig. 5g, h), indicating that the direction of activity change did not affect activation spontaneity. By contrast, in double-chamber nests the lower range of activity levels contained a region where Δ*IoS* > 0 (Fig. 5i, j), indicating a greater bias towards spontaneous activations just before the trough than immediately after it. This result could be explained by two non-mutually exclusive causes: more spontaneous activations before troughs than after, or fewer induced activations before than after. Mapping spontaneous and induced activations separately revealed that both processes contributed to the overall difference (Fig. S14).

In summary, while ants in single-chamber nests tended to activate spontaneously when activity was low, regardless of whether it was rising or falling, ants in double-chamber nests took the direction of the activity change into account, and were more likely to spontaneously activate just before an activity trough. These results suggest that by shielding the inner parts of the nest from external noise, modular architecture enhances the detectability of low-activity states. This may promote spontaneous pacemaker activations immediately prior to the activity trough and hence the initiation of the next activity cycle.

### Tracing activity transmission reveals low-maturity workers are activity leaders

**Fig. 6:**
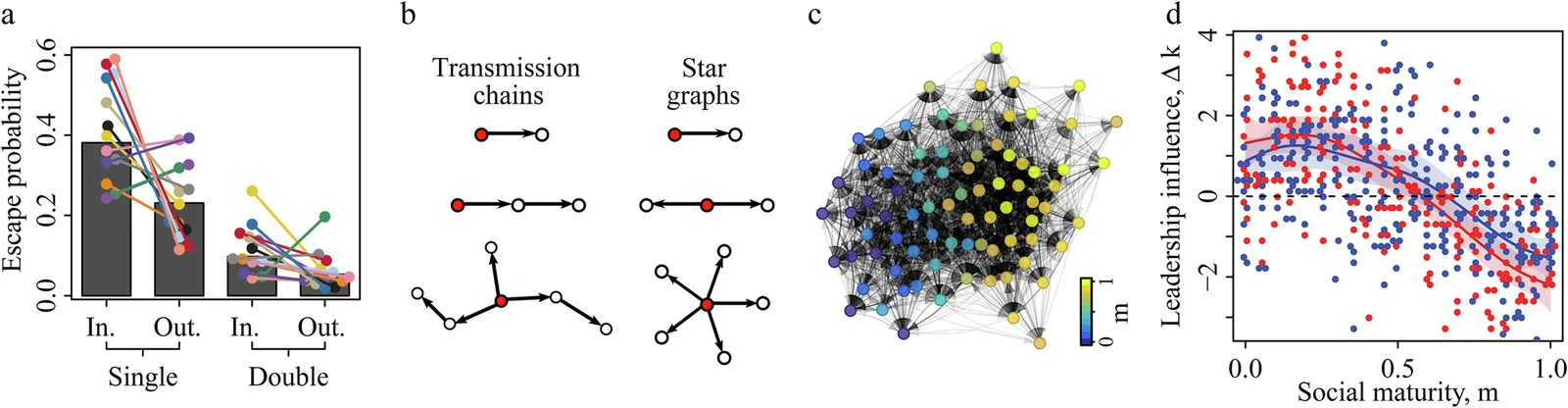
Activity transmission chains. **a** Probability of activity chains escaping from one location to another depending on nest architecture and initiation location (In.: chains initiated in the inner chamber/zone, Out.: chains initiated in the outer chamber/zone). Both main effects were significant (architecture: d.f.=1, *χ*^2^=2079, *p* < 0.0001; location: d.f.=1, *χ*^2^=293, *p* < 0.0001), while the interaction was not (d.f.=1, *χ*^2^=0.18, *p* = 0.67). Escape probabilities were calculated from 88,431 activity transmission chains across *n* = 13 independent colonies and 311 12-hour sessions; points show colony means and bars show grand means. **b** Collapsing transmission chains (left) into star graphs (right). In each transmission chain, the red node denotes the initiator and the white nodes the responders. In the star graphs, directed edges indicate activity transmission from the initiator to all downstream responders. **c** Net activity transmission network for a single 12-hour session (Colony 29, double-chamber nest, day 4). Node colours indicate the ant social maturity, *m*. For every pair of ants *i*, *j*, we identified the overall leader-follower relationship by comparing the number of times *j* acted as a responder on a chain initiated by *i* (*N*_*i*→*j*_) with the number of times *i* acted as a responder on a chain initiated by *j* (*N*_*j*→*i*_). Directed edges point from the net leader to the net follower; for example, if *N*_*i*→*j*_ > *N*_*j*→*i*_, then the edge points from *i* to *j*. Edge shading indicates the absolute difference between the number of times activity was transmitted from *i* to *j*, and the number of times activity was transmitted from *j* to *i*, that is, ∣*ΔN*_*i*↔*j*_∣. Nodes are positioned according to a force-directed layout. **d** Negative association between leadership and social maturity. Leadership was quantified by calculating the difference between the out-degree (i.e., the number of nestmates an ant led, *k*_out_) and the in-degree (i.e., number of nestmates it followed, *k*_in_), *Δk* = *k*_out_ − *k*_in_. Points represent colony means. Red and blue points indicate single- and double-chamber nests, respectively. Curved lines and shaded areas indicate GAMM fits and 95% confidence intervals. The GAMM was fitted to 14,862 ant-level transmission-network observations from *n* = 13 independent colonies, 311 12-hour sessions and 768 ant identities; points show 546 colony-level means.

We next investigated the events following a spontaneous activation, namely, the spread of activity through ant-to-ant contacts^60^. To trace activity contagion through the population of inactive workers, we reconstructed transmission chains initiated by the spontaneous activation of one ant (the initiator), which grew as active ants on the chain successively activated new nestmates (the responders) through physical contacts (see Methods). We then used these chains to explore directional activity transmission between different parts of the nest. Chains initiated in single-chamber nests were significantly more likely to escape from one location to the other than those initiated in double-chamber nests, and furthermore, in both nest architectures chains were more likely to escape from the inner to the outer location than the reverse (Fig. 6a; GLMM with binomial errors, escape probability  ~ architecture  × initiation location, effect of architecture, d.f.=1, *χ*^2^=2079, *p* < 0.0001; effect of initiation location, d.f.=1, *χ*^2^=293, *p* < 0.0001; interaction: d.f.=1, *χ*^2^=0.18, *p* = 0.67). These results provide a mechanistic explanation for our earlier findings that activity coupling is more pronounced from the inner to the outer parts of the nest than in the opposite direction, and that it is weaker in double-chamber than in single-chamber nests (Fig. 4).

We next used the transmission chains to identify influential activity leaders and followers, and to test for an association between leadership and task group. To do so we collapsed each transmission chain into a directed star-graph connecting the initiator to all responders on the chain (Fig. 6b), and aggregated all of the star graphs from a given 12-hour session into an activity transmission network capturing the leader-follower relationship between every pair of ants^62,63^ (Fig. 6c and Fig. S15). We then calculated a leadership score for each ant by calculating the difference between the number of nestmates it led and the number of nestmates it followed. In both nest architectures there was a negative association between leadership and ant social maturity (Fig. 6d; GAMM with a factor-smooth interaction between nest architecture and social maturity; single-chamber nests, estimated d.f.=2.9, F=74.6, *p* < 0.0001; double-chamber nests, estimated d.f.=4.1, F=44.8, *p* < 0.0001). Furthermore, the large overlap between the two fits suggested that the relationship did not depend upon nest architecture. Overall, these results indicate that low-maturity workers are more likely to act as activity leaders that repeatedly initiate activity cascades through the colony and rarely respond to those initiated by others. Reciprocally, foragers are more likely to be reactive followers that readily participate in ongoing activity cascades, but rarely initiate their own.

To test whether the observed negative association between leadership and social maturity was an artefact of the greater numbers of inner-to-outer than outer-to-inner chains (Fig. 6a) and the over-representation of nurses in the inner locations (Fig. 1f and Fig. S10), we repeated the analysis for activity transmission networks constructed from inner-to-inner or outer-to-outer chains only (i.e., chains remaining confined to a single location). Although weaker, the negative associations were still present (Fig. S16), indicating that low-maturity workers are intrinsically more likely to initiate activity chains and hence to act as consistent activity leaders than other ants, irrespective of location.

### Social intermediates act as long-range activity carriers

**Fig. 7:**
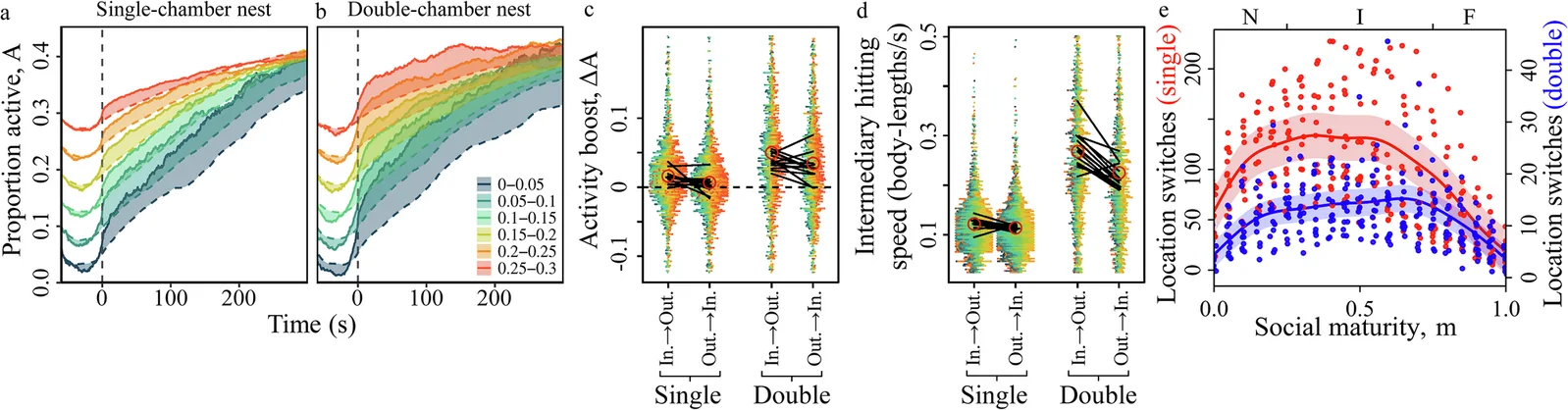
Location-switchers carry activity. **a**, **b** Proportion of ants active during one minute before and five minutes after a contact between an active location switcher and an inactive receiver (solid lines), or in the absence of a location switch (dashed lines), as a function of prior activity levels in the receiving location (colour-coded). Data are shown separately for single-chamber (**a**) and double-chamber nests (**b**). Only the times when the receiving location was quiescent (*A* < 1/3) were included in this analysis. The dashed lines (i.e., in the absence of a location switch) highlight a baseline rise in activity when activity levels are low via reversion to the mean. The solid lines (i.e., in the presence of a location switch) appear consistently *above* the corresponding baseline dashed lines, showing that location switches induce an activity boost (area between the lines, Δ*A*) in the receiving location. The solid-line time series were calculated from 48,556 go-between location-switch events across *n* = 13 independent colonies and 309 12-hour sessions; dashed lines show matched baseline time series without a location switch. **c** Activity boost induced by location switches as a function of nest architecture and switch direction (In. → Out., inner-to-outer; Out. → In., outer-to-inner), calculated over 5 min post arrival. Both main effects were significant (architecture: d.f.=1, *χ*^2^=12.8, *p* = 0.00035; direction: d.f.=1, *χ*^2^=5.5, *p* = 0.019), while the interaction was not (d.f.=1, *χ*^2^=0.0006, *p* = 0.98). **d** Walking speed of the location-switcher, *j*, averaged over the 5 s immediately before contacting the receiver. Small points in (**c**, **d**) represent means calculated for each colony and each pre-arrival activity bin. Open red circles represent grand means. Activity boosts in (**c**) were calculated from 2737 session-level observations across *n* = 13 independent colonies and 309 12-hour sessions. Switcher walking speeds in (**d**) were calculated from 3329 session-level observations across *n* = 13 independent colonies and 309 12-hour sessions. Different letters indicate significant differences in pairwise post-hoc comparisons with Benjamini-Hochberg correction (*p* ≤ 0.0005 for all comparisons). **e** Association between location switching and social maturity. Points represent colony means of the individual-level switch counts during a 12-hour recording session. Red and blue points indicate single- and double-chamber nests, respectively. Single- and double-chamber nests are shown on separate y-axis scales, with the left y-axis corresponding to single-chamber nests and the right y-axis corresponding to double-chamber nests; absolute switch counts should therefore not be compared directly between architectures in this panel. Curved lines and shaded areas indicate GAMM fits and 95% confidence intervals, fitted separately for each nest architecture. The GAMMs were fitted to 16,865 individual-level observations across *n* = 13 independent colonies (8352 observations in single-chamber nests and 8513 observations in double-chamber nests); points show 546 colony-level means. N, I, and F represent the social maturity ranges for nurses, intermediates and foragers, respectively.

We next investigated the mechanisms underlying activity propagation between the inner and outer locations. To do so, we tested the effect of location switchers (i.e., ants that travelled from one location to the other) on the activity levels at the second location, focusing on times when the second location was quiescent prior to the arrival of the switcher to maximise the chance of detecting activity transmission (see Methods). Location switchers consistently precipitated a rise in activity at the second location (Fig. 7a, b, solid lines), and this rise in activity was greater than that observed in a control condition matched for prior activity levels, but with no switchers (Fig. 7a, b, dashed lines). This indicates that relative to the control, location switchers induced an activity boost at the second location (Fig. 7a, b, area between solid and dashed lines), and hence that they served as effective activity carriers. Furthermore, the activity boost depended upon prior activity levels: the more quiescent the second location, the greater the boost (Fig. 7a, b). Interestingly, location switchers induced a greater activity boost in double-chamber nests than in single-chamber nests, and in both nest architectures, the activity boost was greater for inner-to-outer than for outer-to-inner switches (Fig. 7c; LMM, activity boost  ~ architecture  × direction, with colony ID and pre-arrival activity bin as random effects, effect of architecture, d.f.=1, *χ*^2^=12.8, p=0.00035; effect of direction, d.f.=1, *χ*^2^=5.5, p=0.019; interaction: d.f.=1, *χ*^2^=0.0006, p=0.98).

To understand why switches had a greater effect in double-chamber nests, we investigated how the speed of the switcher just before it encountered the receiver (the hitting speed) varied as a function of architecture and switch direction. Switchers attained higher hitting speeds when colonies were housed in double-chamber than in single-chamber nests, and in both nest architectures the hitting speeds were significantly greater for inner-to-outer switches than for outer-to-inner switches. (Fig. 7d; LMM, hitting speed  ~ architecture  × direction, colony ID and pre-arrival activity bin as random effects, interaction direction:architecture, d.f.=1, *χ*^2^=53.99, *p* < 0.0001; post-hoc contrasts with Benjamini-Hochberg correction, effect of architecture: ∣*z*∣ ≥23.86, *p* < 0.0001 for both directions; effect of direction: ∣*z*∣ ≥3.46, p≤0.0005 for both architectures). The correspondence between the activity boost and the hitting speed suggests that the activity induced by a location switch depends upon the excitation of the switcher. To check this, we constructed a mixed model regressing the probability of receiver activation on hitting speed (GLMM with binomial errors & logit link, activation  ~ hitting speed  × architecture, colony ID & Tag IDs nested within colony ID as random effects). This model revealed that while higher hitting speeds increased the likelihood of receiver activation in both architectures (Fig. S17), receivers in double-chamber nests were significantly more sensitive to contacts (*χ*^2^ = 108.2, *df* = 1, *p* < 0.0001). Specifically, for every 1 body-length per second increase in hitting speed, the odds of receiver activation increased 4.29-fold in single-chamber nests, versus a 6.43-fold increase in double-chamber nests. These results suggest that the higher speeds attained by location-switchers and heightened receiver sensitivity compensate for potential structural bottlenecks in modular nests. Together, these factors increase the efficiency of activity transmission, allowing the maintenance of (weak) activity coupling in modular nest architectures.

Finally, we investigated the community affiliations of location switchers. The number of switches per ant depended upon their social maturity, with nurse and forager specialists performing few switches, and intermediates performing many (Fig. 7e, GAMM, switch count  ~ *s*(*m*), single-chamber nests: estimated d.f.=7.74, F=307.30, *p* < 0.0001; double-chamber nests: estimated d.f.=6.57, F=95.96, *p* < 0.0001). Therefore, the transfer of activity from the inner to the outer parts of the nest is largely maintained by intermediate ants rapidly shuttling back and forth, acting as a bridge between the two nest regions.

## Discussion

Overall, our study demonstrates that modular organisation reshapes collective dynamics in ant colonies. By physically compartmenting the workforce, modular nest architecture promotes the coexistence of distinct, locally coordinated activity patterns in different parts of the nest, while compensatory mechanisms help preserve weak but functional coupling across the colony. These findings illustrate how a self-organised system without centralised control solves a challenge common to modular biological systems: how to maintain global coordination in the face of structural fragmentation.

Although the fundamental separation between nurses in the deeper parts of the nest and foragers at the periphery was conserved across architectures, modular nest architecture affected colony organisation at multiple levels. First, it reinforced socio-spatial segregation within the nest and increased social differentiation among workers, in agreement with the prediction that modularity should promote functional diversification between modules^1,2^. Second, modular nest architecture was associated with stronger connectivity *within* groups. According to theory, this could improve information flow and work ergonomics within each group, hence potentially enhancing collective performance by facilitating effective division of labour^1,2^. Furthermore, although modular nest architecture led to predictably weaker ties *between* the two most specialised task groups (nurses and foragers), it also unexpectedly reinforced the strength of connections between these two groups and workers from the intermediate group, hinting at a potential compensatory role of intermediate ants in restoring information flow between spatially-segregated, specialised populations. Finally, nest compartmentation decreased the overall rhythmicity of whole-nest activity cycles, with colonies housed in double-chamber nests exhibiting greater differentiation between a fast, prominent cycle in the inner chamber and a slower, less rhythmical cycle in the outer chamber, suggesting that colonies housed in double-chamber nests function as a weakly coupled oscillatory system.

Quantifying the degree of coupling between activity levels in different parts of the nest revealed the presence of directional inner-to-outer coupling in both nest architectures, although the coupling was weaker in the modular nest. This was confirmed by an individual-level analysis of ant-to-ant activity transmission chains, which showed that in both nest types, chains initiated in the inner location were more likely to reach ants in the outer location than the reverse, and that the proportion of escaping chains was lower in double-chamber nests. Collective activity thus exhibits centrifugal dynamics, originating in the deeper parts of the nest (the pacemaking area) and then spreading out to the periphery. These dynamics echo the hierarchical organisation of other modular oscillatory biological systems, such as peripheral clocks entrained by the central SCN in circadian networks^8,64^, or the cardiac rhythms entrained by the sinoatrial node^13–15^.

Investigating ants’ tendencies to initiate activity revealed a consistent pattern across nest architectures: ants in the inner locations showed a bias toward spontaneous activations, while those in the outer locations exhibited more induced activations. The timing of the spontaneous activations was particularly revealing – ants in both nest types spontaneously initiated activity during low points in the collective cycle, but in the double-chamber nests (and particularly in the inner chamber), ants tended to anticipate the approach of the next activity minimum, spontaneously activating just before activity reached the minimum. This suggests that physical separation enhances each group’s ability to track their position within the activity phase space, free from interference from other groups. These findings align with previous research on *Temnothorax* ants, which found that activity cycles became more prominent when foragers were prevented from returning to the nest, presumably because their irregular returns disrupted the rhythmicity of those that remained in the nest^21^. Our study extends this understanding by demonstrating that while the inner, nurse-dominated location contributes more significantly to pacemaking, ants in the periphery can still track activity fluctuations and generate their own cycles when physically separated. This dual capacity for rhythm generation may represent an important adaptation for maintaining rhythmicity in spatially complex environments.

Comparing how ants’ positions in the colony social network related to their positions in the activity transmission network revealed that irrespective of location, the more socially immature workers (i.e., nurse-like workers) functioned as influential activity leaders, as they were more likely to spontaneously initiate activity chains than to respond to others’ activity. Conversely, socially mature workers (i.e., forager-like workers) acted as reactive followers in both locations, as they were more likely to participate in chains initiated by others than to start their own. This leadership pattern, where the younger workers serve as the primary engine for collective activity, stands in contrast to many mammalian societies, where older individuals typically initiate and lead group movements^65^, and to honeybee colonies, where circadian activity cycles originate from older outside-nest workers^66–68^.

Perhaps more significantly, we also identified a role for socially intermediate workers – ants that were not strongly affiliated with either the nurse or forager communities – in maintaining colony-wide coordination. These individuals frequently switched between the inner and outer locations, and those switches triggered activity rises in the location to which the ant had switched, facilitating activity transmission between nest areas. Importantly, this pattern is consistent with the spatial position of intermediate workers at the interface between the nurse- and forager-dominated regions of the nest. Individuals occupying such intermediate positions are naturally more likely to move between compartments and thus transmit activity between them, suggesting that this ‘messenger’ role may arise from the topology of the colony’s spatial organisation rather than behavioural specialisation. Similar topology-driven propagation has been described in other spatially structured social systems. For example, in giant honeybees (*Apis dorsata*), shimmering defence waves spread across the nest surface through local transmission between neighbouring bees, with individuals located between densely active regions acting as conduits for signal propagation^69^. In our system, this communication channel maintained bidirectional activity flow between nest areas. However, inner-to-outer switches had a greater effect than the reverse, and switching ants were more effective at boosting activity among the receivers in the double-chamber nests. Interestingly, these effects were mirrored by the contact kinetics of coupling encounters: the speed of switchers just before contact was greater for inner-to-outer than outer-to-inner switches, and about twice as high in double-chamber than single-chamber nests, which is likely to explain the greater activity-boosting effect of these switches. The increased movement speed attained by switchers in double-chamber nests could be due to active behavioural modulation by the switchers. Alternatively, it could be a passive consequence of topological differences between the two nest layouts, as switchers in double-chamber nests must travel greater distances and cross areas of lower population density (the connecting passage) than in single-chamber nests, allowing them to attain higher speed. However, regardless of its underlying causes, the increased speed of switchers in modular nest layouts likely plays an important role in restoring partial activity coupling between spatially-segregated, weakly connected parts of the nest by increasing the coupling strength of each connection - not unlike the role played by gap junctions in reinforcing cell-to-cell coupling and hence population-level synchronicity in oscillatory cell circuits controlling the circadian clock^16^ or heartbeat^70^ in mammals, or the role of strong migration flows in synchronising spatially-separated populations showing large-scale prey-predator oscillations^12^.

Overall, our findings provide novel insights into how spatial organisation influences division of labour and collective coordination in modular societies. While modular architecture increases socio-spatial diversity and induces multiple rhythms to coexist within a single colony, the persistence of information flow and activity transmission between physically separated workforces demonstrates the robustness of social coordination mechanisms. This weak coupling between oscillatory populations mirrors patterns seen across biological systems, from neural circuits^71,72^ to ecosystems^12^, implying the existence of common principles underlying coordination in complex modular systems.

## Methods

### Experiments

Thirteen queenright *L. acervorum* colonies were collected in Anzeindaz, Switzerland in June 2017 (range 36-104 workers, see Table S1), then kept under controlled laboratory conditions until the beginning of the experiments. Experimental nests were constructed by sandwiching a 2 mm-thick rectangular cardboard gasket between two microscope slides. The single-chamber nest consisted of a single room (63 × 42 mm), while the double-chamber nest was divided into two equal rooms (63 × 21 mm each) connected by a narrow passage (2 × 2 mm cross-section, 5 mm long; Fig. S1a-b). This passage served as a structural bottleneck for inter-chamber movement. Both architectures provided an identical internal area (2646 mm^2^) and a single entrance leading to the foraging arena where protein (*Drosophila*), dilute sugar water, and drinking water were provided ad libitum.

Colonies were pseudo-randomly allocated to their starting nests in a paired crossover design (Fig. S1c). To ensure that colony demographics were comparable between the two starting architectures, colonies were ranked by size and assigned to their initial architecture in a staggered fashion (Table S1). Seven colonies began the experiment in the single-chamber nest (mean *N* adult ants: 53 ± 6, brood: 131 ± 18), while six began in the double-chamber nest (mean *N* adult ants: 61 ± 10, brood: 128 ± 19).

Two days before the beginning of the experiment, all ants in a colony were individually marked by gluing a paper fiducial marker (0.7 × 0.7 mm; ARTag library^73^) to the dorsal surface of their thorax using Pattex Power Easy Gel superglue. Ants were temporarily immobilised via a brief carbon dioxide bath for the duration of the procedure. Once tagging was complete, a final census of ants and brood was performed.

The colonies were then transferred into their allocated starting nest and left for 24 hours to habituate before tracking was started. Each colony was then continuously tracked for six days (replicate 1; Fig. S1) using a custom-built automated ant tracking system^50^ (see below). After the first six-day tracking session (Replicate 1), tracking was stopped and the colonies were moved out of their experimental nests to allow re-tagging of ants that had lost their tags. If an ant’s identity could be confidently ascertained (e.g., via unique body marks or injuries), the original barcode ID was maintained; otherwise, a new unique barcode was assigned. After the re-tagging period (median 23 hours), colonies that had initially been in the single-chamber nests were moved into a new double-chamber nest, while those that had been in double-chamber nests were moved into a single-chamber nest (Fig. S1). Colonies were then left for a 24-hour habituation period before starting the second six-day tracking session (Replicate 2).

Across both replicates, automated tracking was conducted using an infra-red-sensitive video camera (4560 × 3048 pixel resolution) operating at two frames per second^50^. To ensure the nest appeared dark to the ants – which are insensitive to infra-red light^74^ – an infra-red-passing filter (640 nm, R-64, Hoya, USA) was placed over the nest roof. Throughout the 12 days of tracking, colonies were maintained on a 12:12 h diurnal cycle (25^∘^C with visible light from 07:30-19:30; 20^∘^C in darkness from 19:30-07:30). After the experiments, spatial trajectories were extracted from the tracking data and post-processed using an open-source pipeline^47,52^. Physical contacts were inferred using a geometrical approach^47,50,52^ in which each ant was represented by a trapezoid aligned with its body axis and extending from the antennae tips to the end of the gaster. A contact was recorded when the front end of one ant’s trapezoid fell within that of another.

### Defining bouts of ant activity and inactivity

To divide worker trajectories into discrete sequences of homogeneous segments, each associated with an activity state, we followed an existing two-stage method^49^ (Fig. S9). First, trajectories were segmented at ‘change points’, corresponding to conspicuous changes in movement characteristics. Trajectory segmentation was carried out using the *changepoint* package version 2.2.2^75^ for *R*, which identified the points at which the instantaneous speed (Fig. S9a) exhibited a change in both the mean and the variance^76^. The trajectory segments separated by these change-points are referred to as ‘bouts’.

Second, we carried out principal components analysis and cluster analysis to assign a label to each bout. To that end, each bout was characterised using seven statistics, namely (i) the mean, standard deviation and coefficient of variation of the speed, (ii) the mean, standard deviation and coefficient of variation of the unsigned turn angle, and (iii) the duration of the trajectory segment. We then pooled the summary statistics for all trajectory segments across all individuals within each colony, and subjected these pooled multi-dimensional data to a principal components analysis. Scatterplots of the first and second principal components showed the same L-shaped distribution across all colonies (Fig. S9b). As this was suggestive of two categories, we subjected the first two principal components of all bouts in a colony to a Model-Based Clustering using the Mclust function from the *mclust* package for R^77^, with the number of clusters set at 2. The resulting binary classification scheme allowed each bout to be assigned to one of two categories: one consisting of short periods of fast and straight motion with high variation in speed but low variation in turn angle, which we labelled ‘active’ (i.e., consistently moving), and another consisting of longer periods of slow and tortuous motion, with low speed variation but higher turn angle variation, which we labelled ‘inactive’ (i.e., stationary). These classifications were robust, as in all colonies the inactive bouts were always associated with lower speeds and larger turn angles (Fig. S9c, d).

### Spectral analysis

The individual-level activity time series were used to calculate the collective activity levels. For each frame within a given 12-hour observation period, we calculated the proportion of the ants currently within a given area (i.e., the whole nest, or a specific nest location) that were in the active state. As the tracking camera operated at 2 frames/sec, each 12-hour collective activity time series comprised 86’400 observations. Following Doering et al. (2024), a symmetrical 240-second-wide Gaussian-weighted smoother was applied to each time series to filter out high-frequency noise. We then used Wavelet analysis to construct the power spectrum (i.e., the periodogram) for each time-series^22^. The dominant cycle *λ* was defined by the tallest peak in the periodogram, and the rhythmicity *β*, was defined as the ratio of the power associated with the peak period versus the mean power across all other periods^25^.

### Measuring social maturity and determining the number of worker task groups

The topological position of each ant in the colony contact network was used to quantify its social maturity, based on an existing method^55^. All the ant-to-ant contacts within a given 12-hour session were used to construct a time-aggregated contact network, resulting in 311 contact networks in total (Fig. S2-S3). Nodes in these networks represented individual ants, and weighted edges represented the number of contacts between each pair of ants. A soft community detection algorithm (Facetnet^53^) was then applied to each network to identify overlapping worker communities, that is, clusters of workers that more frequently contact fellow group-members than workers in other groups. We first used modularity maximisation to estimate the optimal number of communities in the 12-hour contact networks. To do so, we ran Facetnet 1000 times on each 12-hour contact network, doing so for either two or three communities. For each network, we then identified the two- and three-community allocations that achieved the greatest modularity, thus producing two solutions per network. Comparing the modularities associated with the two- and three-community solutions revealed that the modularity was significantly greater for the two-community solutions (Fig. S4, LMM, modularity  ~ number of communities, with colony identity and 12-hour session identity as random effects, one model per nest architecture). Therefore, as in several other species of ant^55–57^, the *Leptothorax acervorum* contact networks were best described as two distinct but partially overlapping communities, that is, the nurses and the foragers (Fig. S4). As Facetnet assigns continuous scores in the range 0-1 reflecting the affiliation of each individual to each community, we used the scores for the forager community to quantify the social maturity, *m*, of each worker^55–57^. Following Richardson et al. 2021, wherever an analysis involved the use of categorical communities, any ants (or sites) with *m* < 1/4 were labelled nurses (or nurse-sites), and any ants (or sites) with *m* > 3/4 were labelled foragers (or forager sites). Using more restrictive thresholds did not qualitatively affect the results.

### Using social maturity to identify inner and outer spatial zones in single-chamber nests

The spatial preferences of each ant were quantified using the approach of Richardson et al. (2022)^49^. Nests were first discretised into a regular hexagonal lattice overlaid across the entire nest footprint (where the width of one hexagon was set to 1/2 the worker body length). As both nest geometries had the same total area, this allowed spatial comparisons to be made at equivalent spatial resolution within and between chambers. The number of visits by ant *i* to each site *s*, ${n}_{i}^{s}$, was then counted. These counts were then used to calculate the weighted mean social maturity of the typical visitor to a given site, that is, the site maturity,

$${m}^{s}=\frac{\mathop{\sum }_{i = 1}^{I}{n}_{i}^{s}\cdot [{m}_{i}]}{\mathop{\sum }_{i = 1}^{I}{n}_{i}^{s}}$$

The weighting ensures that the contribution that an individual *i* makes to the calculation of the social maturity of the typical visitor to a given site, is proportional to the number of visits it made to *s*. In all 13 colonies, workers with low social maturity (i.e., the nurses) tended to frequent areas deep within the nest, whereas those with high social maturity (i.e., nest guards and foragers) frequented sites near to the nest entrance (Fig. S6-S7).

In order to identify distinct inner and outer zones for colonies housed in single-chamber nests, we used the median of the *m*^*s*^ distribution for a given 12-hour session. Sites with *m*^*s*^ below the median (i.e., those visited mainly by nurses) were defined as belonging to the inner spatial zone, and those above it (i.e., those visited mainly by foragers) defined as belonging to the outer spatial zone (Fig. S6).

### Granger causality testing

We used linear Granger causality testing to measure directional coupling between the collective activity in the inner and the outer locations. To do so the activity time-series for the inner and outer locations were split according to the 12-hour day/night sessions, and the mean activity calculated once per minute. We then used the causality.test function from the NlinTS package^78^ (version 1.4.5) for R, with a lag of 2 minutes, to test for Granger causality between the inner and outer time-series.

The Granger causality test results depended upon two parameters: the width of the time bin used to coarse-grain the inner and outer activity time series, and the lag parameter used in the test. To check that our results were not contingent upon the precise parameter choices for the time-series bins or the lags, we repeated the analyses for 51 additional combinations of time bin and lag, and then separately fitted binomial GLMMs with nest architecture and causal direction as additive fixed effects, and colony, session number and day/night as random effects, to the binarised Granger test p-values from each parameter combination. The sign of the coefficients was mostly unaffected by the time bin and lag parameters (Fig. S11).

### Social contagion of activity but not inactivity

To investigate whether worker activity (and inactivity) spread by social ‘contagion’, we tested for statistical associations between activation and deactivation, and the recent history of contacts with active or inactive nestmates. In order to detect activity contagion, we focused on ants that were inactive at times when the whole-colony activity time-series was in a local ‘trough’ (Fig. S12a, b). Then, for each time-step during the trough, we recorded the number of encounters that the focal ant had with both active and inactive nestmates during the previous 10 seconds, and also whether it activated during the next 10 seconds (Fig. S13a, Table S2). Similarly, to detect contagion of inactivity, we focused on ants that were active when the colony activity was at local peak, and examined whether the number of previous encounters with active and inactive nestmates influenced the likelihood that the ant subsequently deactivated (Fig. S13b, Table S2).

### Quantifying activity cycle pacemaking

To categorise each activation as either spontaneous or induced, we used the number of contacts with active nestmates that the focal ant experienced during a 60 s window preceding the activation. If the focal ant experienced at least one contact during the window, the activation was labelled *induced*, whereas if there were no contacts, it was labelled *spontaneous*. Furthermore, to ensure that this analysis focused on behaviourally meaningful activations, we only considered activations in which the focal ant was inactive for at least 90% of the preceding 1-minute window.

To quantify the tendency of an ant to activate spontaneously, we expressed the number of spontaneous activations as a fraction of the total number of activations, *IoS* = *n*_*S*_/(*n*_*S*_ + *n*_*I*_). As *IoS* is a proportion, values close to 1 indicate mainly spontaneous activation, and values close to 0 indicate mainly induced activation. As each worker typically performed both spontaneous and induced activations when activity was low or high, and when activity was rising or falling, and as activation spontaneity varied across space, a unique *IoS* was defined for each individual at each position in the activity phase space and in each location (outer vs. inner).

### Semi-parametric modelling of activity pacemaking

Generalised additive mixed modelling (GAMM)^79,80^ was used to quantify how the current activity level and the (signed) activity slope predicted the *IoS*. Rather than using the whole-nest time-series, the activities and slopes were defined by the location of each activation, so an activation that occurred in the inner chamber was assigned the current activity level and slope from the inner chamber. Activity and slope were computed from the smoothed time-series, obtained by applying a moving average with a trailing window spanning *t* − 30 to *t* seconds to the raw activity data (Fig. 5a).

Ants typically performed 83 ± 5 (grand means and S.E., N=13 colonies) activations within a given 12-hour session, doing so for multiple combinations of the current activity level and activity slope. Therefore, the dependent variable, *IoS*, was calculated once for each activity level-slope combination at which an ant performed at least one activation.

To test whether *IoS* was best predicted by activity level alone, activity slope alone, or both together, we constructed four GAMMs using the function *bam* from the *mgcv* package (version 1.8-31) for R^81^. Semi-parametric models consist of parametric and non-parametric (smoothed) terms. In all models the parametric terms consisted of an intercept, a categorical main effect for the location of the activation, and another for the nest architecture. The four models differed in the non-parametric smooth terms. Model 1 included a smooth term for activity level only, and model 2 included one for activity slope only. Model 3 included two smooth terms, one for activity level and another for activity slope. Model 4 included a tensor-smooth term modelling the combined influence of activity level and activity slope. Comparing the parsimony of the different models revealed that *IoS* was best described by model 4 (Tables S3-S4; Fig. 5).

All models also included four random effects (colony and ant identity, 12-hour session number, and day/night) in the non-parametric part of the model using the bs=‘re’ arguments for *mgcv*. To capture the uneven distribution of on-switching by different ants across the activity phase space, the counts of the number of activations that an ant made for each activity-slope combination were supplied as the ‘weights’ argument in each model. To ensure fair comparisons between models, all smooth terms used thin-plate splines, and 30 knots. In all models the ‘by’ argument in *mgcv* was used to obtain a factor-smooth for each of the four architecture-location combinations.

As *IoS* is a proportion, all models used a binomial error distribution and a logit link function. Because binomial models produce coefficients which are logarithms of odds ratios (i.e., $\beta =\ln [p/(1-p)]$), to aid interpretation, the log-odds were back-transformed into probabilities using the inverse logit function, *p* = *e*^*β*^/(1 + *e*^*β*^). The activity heatmaps (Fig. 5) show these probabilities. The contours plotted in Fig. 5 were obtained using the package *itsadug*^82^ (version 2.3) for R, to obtain 95% simultaneous confidence intervals for the fitted values.

### Activity transmission chains

To identify an activity transmission chain, we first identified all induced activations falling within a 5-minute forward window following each spontaneous activation. As induced activations were always preceded by one or more contacts with active nestmates, we then compiled a list of the active-nestmate contacts associated with the induced activations in the window, including their start and stop times and the identities of the ants involved. We then searched within the list for the shortest time-respecting paths^83^ connecting the ancestral spontaneous activator (the initiator) to the descendant induced activators (the responders; Fig. 6).

To maximise the chances that the transmission chains capture activity transmission, we implemented two filtering steps. The first excluded any activations in which the activating ant had already been active more than 10% of the time during the previous minute, thus ensuring that the activations that form the links in the chain represented biologically meaningful changes in behaviour. The second filter excluded any activation in which the mean activity level of the donor during the period between its own activation and its encounter with the receiver fell below 90%, thus ensuring that the activity state of each chain member could be traced back, through an uninterrupted sequence of continuously active intermediaries, to a single individual – the initiator.

### Quantifying pairwise leader-follower relations

Each activity chain was collapsed into a star graph connecting the single initiator to all responders on the chain. All the activity transmission star graphs observed for a given 12-hour session were then aggregated to construct a raw activity transmission network. Each unique ant pair {*i*, *j*} in these raw transmission networks were connected by two directed and weighted edges; the weight assigned to the edge pointing from *i* to *j* gave the number of star-graphs in which *i* was the initiator (hub) and *j* a responder (spoke), that is, *N*_*i*→*j*_, and the weight assigned to the *j* → *i* edge gave the number of star-graphs in which *j* was the initiator and *i* a responder. Finally, to produce the final net activity transmission network, edges in the *i* → *j* and *j* → *i* directions were collapsed into a single directed edge, by calculating the signed difference between the weights,

$$\Delta {N}_{i\leftrightarrow j}={N}_{i\to j}-{N}_{j\to i}$$

The sign of this difference was used to identify the net leader and follower in each pair^62^. Thus, an edge was drawn from *i* to *j* when Δ*N*_*i*↔*j*_ > 0, whereas when Δ*N*_*i*↔*j*_ < 0 an edge was drawn from *j* to *i*. If the difference was zero, no edge was drawn.

### Measuring activity changes associated with location switching

To assess the transfer of activity associated with location-switching ants, we first identified occasions when a physical contact between two ants *i*, *j* within a given location, was followed by one of the pair, *j*, travelling to the other location where it contacted an inactive ant, *k*. If location-switching ants serve as activity carriers, we expected the arrival of the switcher, *j*, to be followed by a rise in activity among the ants at the second location. To maximise the chances of detecting activity transmission, we focused on location switches that met three conditions, namely, (i) the switcher *j* arrived in a quiescent location, that is, one in which the mean proportion of ants that had been simultaneously active during 1 minute immediately preceding the *j*, *k* contact was less than 1/3, (ii) the receiver *k* was inactive prior to the contact with the switcher, and (iii) the switcher was active for at least 90% of the time between the *i*, *j* and *j*, *k* contacts. As the activity-matched controls quantified the activity change at a given location after a quiescent period when no switches occurred, and as activity levels following such a period will rise just by reversion to the mean, we defined the activity boost as the average difference between the activity after a location switch, and after the activity-matched control, Δ*A* = *A*_*switch*_ − *A*_*noswitch*_.

### Influence of hitting speed on receiver activation

To assess the influence of hitting speed on receiver activation, we quantified the behavioural outcomes of contacts between active and inactive ants. We utilised two 10-second temporal windows: (i) a ‘past’ window immediately prior to the start of the physical contact, used to classify the initial states of each ant and the mean hitting speed of the sender, and (ii) a ‘future’ window immediately after the contact start, used to identify receiver state transitions. A contact was only included in the analysis if both ants had at least 75% tracking coverage during the past window, and the same in the future window. We focused only on active-inactive contacts, that is, any encounter in which one ant spent  > 90% of the past window in the active state and the other spent  < 10% active. Receiver behaviour was scored as a binary response variable, with contacts in which receivers transitioned from inactive to active scored as 1, and those in which the receiver remained inactive scored as 0. We then analysed receiver activation using a generalised linear mixed model with binomial errors and a logit link function, including hitting speed, nest architecture and their interaction as fixed effects, and colony identity and ant identities nested within colony identity as random effects (Fig. S17).

### Reporting summary

Further information on research design is available in the Nature Portfolio Reporting Summary linked to this article.

## Supplementary information

Transparent Peer Review file

Supplementary Information

Description of Additional Supplementary Files

Supplementary Data

Reporting Summary

## Data Availability

The raw data used for the main analyses described in this study are available at https://doi.org/10.5281/zenodo.15020312^84^. Source data underlying the graphs and charts presented in the main figures are provided in a Supplementary Data file, with separate worksheets for each figure panel or analysis.

## Code availability

The software pipeline used for the main analyses described in this study is available at https://doi.org/10.5281/zenodo.15020312^84^.
