# Supplementary material for: Modular nest structure influences activity cycle synchronisation in ant colonies
Source: Commun Biol. 2026 Jun 27;9:1317. doi: 10.1038/s42003-026-10560-x (PMC13649663; doi:10.1038/s42003-026-10560-x)
Supplement: Supplementary file 1 [file 42003_2026_10560_MOESM1_ESM.pdf]

nature portfolio

Peer Review File

Modular nest structure influences activity cycle synchronisation in ant colonies

Corresponding Author: Dr Thomas Richardson

This file contains all reviewer reports in order by version, followed by all author rebuttals in order by version.

Attachments originally included by the reviewers as part of their assessment can be found at the end of this file.

Version 0:

Reviewer comments:

Reviewer #1

(Remarks to the Author)
Richardson and colleagues study spontaneous activity cycles in Leptothorax ant colonies. They find that these cycles are driven by nurses acting as pacemakers, that their activity is contagious, that nurse-forager intermediates transfer activity to foragers to synchronize the colony, and that modular nest architecture reduces synchrony.

Overall, this is a very good manuscript. The analyses are innovative, rigorous, and well-presented, and the story is reasonably linear and the major results well supported. I generally enjoyed reading this paper. I have only minor comments and questions, which I hope will improve the manuscript. While the authors should feel free to add analyses that answer these, I expect that most can be addressed without additional work.

- it would help to have a schematic of the experimental design and nest architecture
- maybe i missed this, but what 'social maturity' thresholds do you use to define nurses and foragers? it would also help to show this as a figure panel somewhere - perhaps as worker type (N,I,F) vs social maturity
- multiple central claims in the paper rest on the argument that these colonies are best represented by two interaction clusters (i.e. nurses and foragers). would your results in Fig. 2 and 3 change if you have five or seven or some other large number of clusters instead?
- on a related note, what proportion of nurses act as pacemakers? are all nurses capable of initiating activity spontaneously? are there specialist pacemakers within the colony?
- does a single ant typically function as the colony pacemaker at any one time? for the mean colony activity cycle, how many pacemakers independently initiate activity? how does this vary with colony size?
- what do the trajectories of pacemaker ants look like within the nest? do they wander around until they bump into their neighbors? i understand there is no evidence for negative feedback when they contact a stationary neighbor: do the pacemakers just run around for the duration of activity cycle? or do they stop early?
- how is the number of contacts between ants defined? how sensitive are your results to error in this classification?
- does the 'hitting speed' of an individual switching ant correlate with the activation probability of its (individual) target? this seems important to support the claim in Fig. 7c,d.
- some of the language (eg. line 217) implies that pacemaker ants actively track colony activity cycles to decide when to spontaneously activate. the analyses presented do not explicitly show this; I would edit the text for accuracy
- different figures use different notation that is slightly confusing: eg. I for intermediate vs I for inner.
- please check for typos and consistency: eg some panels are missing labels and significance markers, some references are formatted incorrectly (eg line 388)

Reviewer #2

(Remarks to the Author)
This manuscript presents an elegantly executed analysis of collective activity dynamics in Leptothorax acervorum colonies across single- and double-chamber nest architectures. The dataset is impressive in scale, the tracking quality is high, and the multi-layered analytical pipeline—combining contact networks, spectral characterization, activation dynamics, and transmission-chain inference—is sophisticated and innovative. The question of how modular nest structure affects collective

oscillations of ants' activity is biologically meaningful and potentially inspiring for other systems. The core results regarding oscillatory dynamics and the spatial modulation of activity waves are compelling.

However, I have several substantive concerns about the interpretation of the results—particularly regarding (i) the role of “nurses” in initiating activity cycles, and (ii) the proposed function of “intermediates” as activity transfer specialists. These concerns arise from how community affiliation, spatial structure, and task categories interact in the analytical pipeline. Addressing the points below will substantially strengthen the manuscript’s conceptual clarity and generality.

1.The maturity axis and task groups depend strongly on the manual choice of community number in FacetNet. FacetNet requires the user to predefine the number of communities (K). Since the authors chose K = 2, the resulting “social maturity” score necessarily represents a projection onto two user-specified community poles. All workers must therefore lie somewhere on this single axis, regardless of whether the natural structure of the colony is bimodal, trimodal, or continuous.

The subsequent definition of three behavioral groups (nurses, intermediates, foragers) emerges from thresholding of this continuous 0–1 maturity score, rather than from FacetNet itself. The maturity distribution is bimodal, not trimodal, so the intermediate group arises from spatial gradients in contact patterns rather than from a formally detected community.

Because many downstream analyses—including leadership, switching behavior, and activation patterns—use these categories, the conclusions about "distinct nurse and intermediate roles" may partially reflect the choice of K = 2 and threshold placement rather than emergent biological groupings.

2. Leadership varies smoothly across maturity, complicating categorical interpretations. Figure 6d shows leadership is flat from m = 0 to 0.5. Thus, a substantial number of early-intermediate individuals exhibit leadership behavior, even though the manuscript categorizes them as non-nurses. This creates a mismatch between the continuous maturity metric and the categorical interpretation that "nurses initiate activity cycles."

The authors might consider rephrasing this conclusion more broadly (e.g., “low-maturity individuals”), or analyzing initiation rates as a function of continuous maturity.

3: Spatial adjacency may underlie the observed “messenger” role of intermediates. Many of the observed “messenger-like” behaviors (high switching frequency, bridging inner and outer regions) appear consistent with intermediates' spatial positions—they occupy the region between the nurse- and forager-dominated zones. In any spatially structured excitable system, units located between two dense compartments will naturally serve as conduits for propagation. This suggests that intermediates' apparent transfer role may arise from topology rather than specialized task differentiation. This pattern is conceptually similar to how Giant honeybees spread shimmering vibration waves across the nest.

4: The definition of “activity phases” (low/high) need to be clarified. The four activation-phase categories (low–rising, low–falling, high–rising, high–falling) play an important role in interpreting spontaneous activations. While the slope definition is clear, the threshold separating "low" from "high" activity is not explicitly defined in the Methods. This makes it difficult to assess or reproduce the phase-specific findings in Figure 5.

5: Potential circularity between spatial structure and inferred behavioral roles was not addressed. Community affiliation, maturity, inner/outer zoning, leadership, switching rates, and propagation patterns all derive from the same spatially structured trajectory data, there is a possibility of circular inference:
1. Spatial layout determines contact network structure
2. Contact network determines "maturity"
3. Maturity determines task labels (nurse/intermediate/forager)
4. Spatial measures then correlate with these labels
5. Conclusions treat these labels as independent behavioral roles

Reviewer #3

(Remarks to the Author)
In this manuscript, the authors thoroughly examine social network dynamics related to activity rhythms in nests of Leptothorax acervorum. They focus on comparing these dynamics in one-chamber vs. two-chamber artificial nests. They observe 13 colonies that are each housed in both one-chamber and two-chamber nests (for paired analysis) and are filmed continuously. Analyses include identifying the spatial location of rhythm-setting activity (inner areas), which behaviorally specialized workers tend to initiate activity (nurses), where behaviorally specialized workers tend to be located in colonies (nurses towards the inside), and how activity is coordinated among locations and chambers (e.g., less coupled in multi-chamber nests). The primary method used is automated tracking of individuals and classification of social interactions. I appreciate the pairing of manipulative/artificial aspects (namely the nest architecture) with fairly non-invasive monitoring of natural behavioral dynamics in this species. The authors identify the broader significance of this study as relating to oscillations and coordination between different oscillating modules, recurring phenomena in nature.
I find this study to be thoughtful and detailed. I think the references are appropriate and I believe this study is original. I have no major complaints about the methods or interpretations, but I have a few smaller comments mainly related to improving clarity.

Detailed comments:
Line 25: I think the em dash spacing needs to be fixed.
Line 29: Should this be “turn” singular? Not sure of the intended grammar.
Line 30: Consider removing “the” before METs.
Line 71: Please provide more clarity here (rather than just in the method) about the six-day time period vs. the 12-hour filming periods.
Line 77: From the description of the artificial nests, it seems like the top portions would be clear glass. While this light/dark regimen is fairly standard, would having ants that otherwise nest inside of acorns or rock crevices be exposed to 12 hours of direct light possibly influence behavioral rhythms?
Line 88: Consider rephrasing for clarity; since each colony was house in both one- and two-chamber nests, does it make sense to phrase it as “these nests contained more fully specialized workers” when they are the same workers as before? Maybe you mean the ants acted in more specialized ways in this environment.
Line 95: Please specify the grid size here.
Line 100: How was the grid done for two-chamber nests to allow for comparison between the two chambers?
Line 169: I would mention your threshold for determining inactivity here, not just in the methods.
Line 253: Please clarify why the choice was made to focus on times when the second location was quiescent prior to switcher arrival.
Line 282: Somewhere in the discussion I would really like to see information on the relationship of your results to real Leptothorax acervorum biology/nests. Given that they live in pre-existing structures that seem to typically have only one large interior space, do they ever take steps to subdivide them into chambers?
Line 387: Fix this reference.
Line 514: Is the R code also available somewhere?

Version 1:

Reviewer comments:

Reviewer #1

(Remarks to the Author)
The authors have satisfactorily addressed my comments, and I find the revised manuscript improved - and suitable for publication.

Reviewer #2

(Remarks to the Author)
The authors have addressed all of my major concerns and have revised the manuscript thoroughly. The updated figures' captions and clarified methodologies have significantly strengthened the paper. I am satisfied with the current version and recommend it for publication in its present form.

Open Access This Peer Review File is licensed under a Creative Commons Attribution 4.0 International License, which permits use, sharing, adaptation, distribution and reproduction in any medium or format, as long as you give appropriate credit to the original author(s) and the source, provide a link to the Creative Commons license, and indicate if changes were made.
In cases where reviewers are anonymous, credit should be given to 'Anonymous Referee' and the source.
The images or other third party material in this Peer Review File are included in the article's Creative Commons license, unless indicated otherwise in a credit line to the material. If material is not included in the article's Creative Commons license and your intended use is not permitted by statutory regulation or exceeds the permitted use, you will need to obtain permission directly from the copyright holder.
To view a copy of this license, visit https://creativecommons.org/licenses/by/4.0/

Reviewer #1 (Remarks to the Author):

Richardson and colleagues study spontaneous activity cycles in Leptothorax ant colonies. They find that these cycles are driven by nurses acting as pacemakers, that their activity is contagious, that nurse-forager intermediates transfer activity to foragers to synchronize the colony, and that modular nest architecture reduces synchrony.

Overall, this is a very good manuscript. The analyses are innovative, rigorous, and well-presented, and the story is reasonably linear and the major results well supported. I generally enjoyed reading this paper. I have only minor comments and questions, which I hope will improve the manuscript. While the authors should feel free to add analyses that answer these, I expect that most can be addressed without additional work.

We thank the reviewer for their positive assessment of our study. We are encouraged by their view that our analyses are "innovative, rigorous, and well-presented" and that the major results are well supported. We also appreciate the reviewer's assessment that most of the remaining points can be addressed without additional analyses, and we have revised the manuscript accordingly to improve clarity and conceptual framing.

- it would help to have a schematic of the experimental design and nest architecture

We thank the reviewer for this suggestion. We agree that the crossover design may be difficult to visualise. We have therefore added a new schematic to the Supplementary Information (Fig. S1) illustrating the nest architecture and crossover design. We have also revised the relevant section in the Results (lines 71-83) for clarity.

- maybe i missed this, but what 'social maturity' thresholds do you use to define nurses and foragers? it would also help to show this as a figure panel somewhere - perhaps as worker type (N,I,F) vs social maturity

Following Richardson et al. (2021), we defined nurses as individuals with a social maturity score m < 0.25, foragers as those with m > 0.75, and intermediates as those with 0.25 ≤ *m* ≤ 0.75. The thresholds are described in the Methods section, 'Measuring social maturity' (lines 433-435). We have now also updated Figure 1 to indicate these cutoffs on the social maturity distributions.

- multiple central claims in the paper rest on the argument that these colonies are best represented by two interaction clusters (i.e. nurses and foragers). would your results in Fig. 2 and 3 change if you have five or seven or some other large number of clusters instead?

The choice of K = 2 communities was not arbitrary. In previous work across several ant species, we systematically compared solutions with varying numbers of communities and found that K = 2 consistently maximised modularity, with modularity decreasing as additional communities were imposed (Richardson et al., 2021; Kay et al., 2023). We adopted the same approach here. As shown in Figure S4, the two-community solution yields higher modularity than solutions with K = 3, indicating that K = 2 provides the best-supported partition of the *Leptothorax acervorum* contact networks. We therefore concluded that a two-community model

offers the most parsimonious and biologically meaningful representation of these colonies and based all subsequent analyses on this partition. This rationale is noted in the Methods section, 'Measuring social maturity' (lines 428-430).

- on a related note, what proportion of nurses act as pacemakers? are all nurses capable of initiating activity spontaneously? are there specialist pacemakers within the colony?
- does a single ant typically function as the colony pacemaker at any one time? for the mean colony activity cycle, how many pacemakers independently initiate activity? how does this vary with colony size?

Thank you for raising these interesting questions. To better characterise pacemaking, we conducted new analyses of spontaneous activations occurring within the 60 s preceding an activity trough ("pacemaking events").

We first quantified how many ants spontaneously activated before each trough. Across the 26,910 detected troughs, this yielded a mean of 2.5 ± 0.2 ants per trough (mean ± SE). The number of spontaneously activating ants per trough increased significantly with colony size (GLMM with colony identity, replicate, and recording session as random effects, effect of colony size: χ^2= 175.67, df=1, P<0.0001), ranging from 1.85 ants per trough in the smallest colonies (N=33 ants) to 5.3 ants per trough in the largest colonies (N=104 ants).

We next calculated, for each 12-hour session, the proportion of nurses that exhibited at least one pacemaking event. This proportion was consistently high (mean ± SE = 0.94 ± 0.004 across sessions; Fig. R1a), indicating that pacemaking is broadly distributed among low-maturity workers rather than being restricted to a small subset of individuals.

However, nurses differed in how often they contributed. The distribution of pacemaking events per nurse was right-skewed (Fig. R1b), indicating that some individuals contributed more frequently than others.

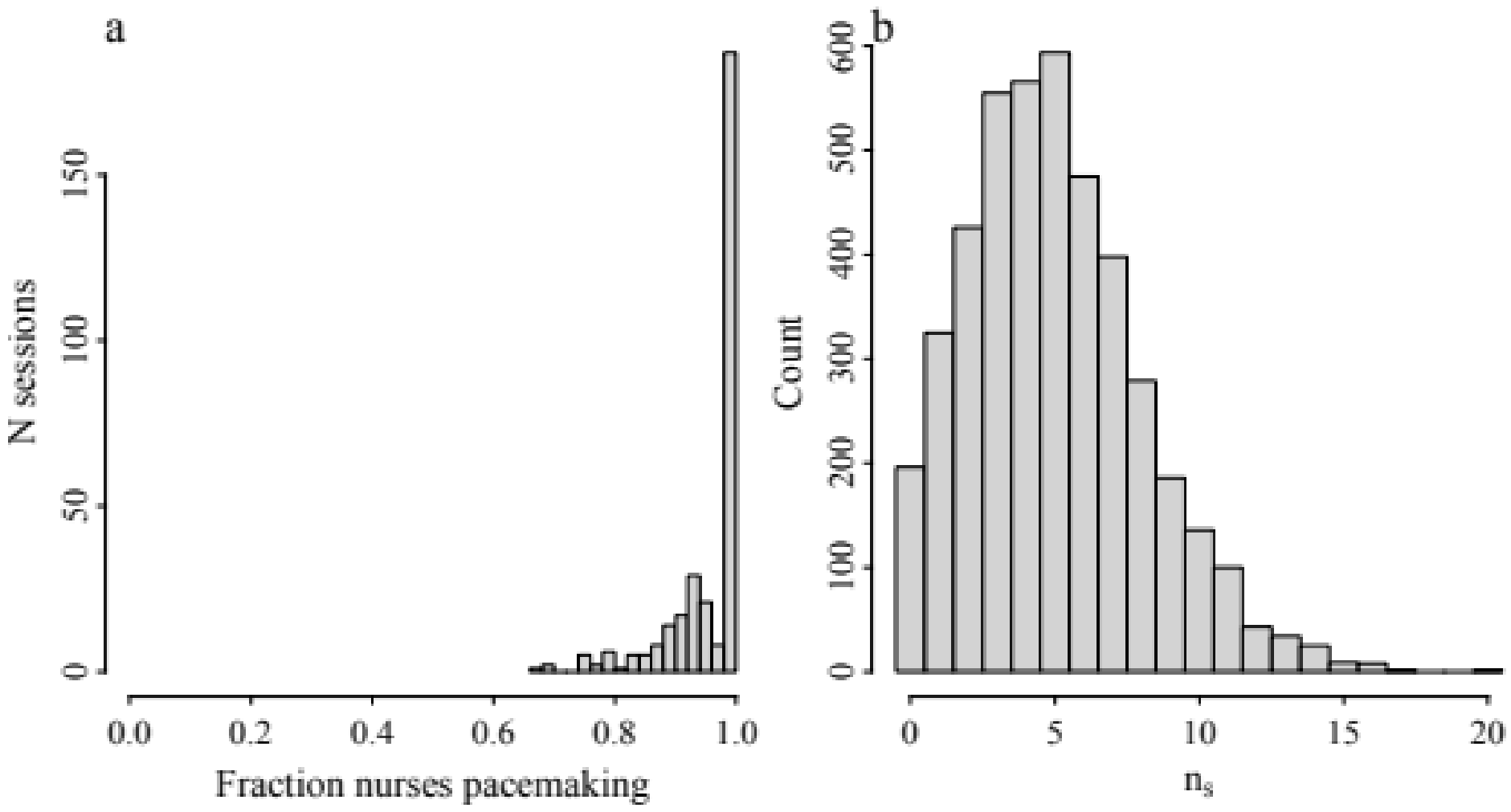

Figure R1. Distributions of pacemaking activity among nurses. (a) Frequency distribution of the proportion of nurses per 12-hour session that exhibited at least one spontaneous activation within the 60 s preceding an activity trough. (b) Frequency distribution of the number of spontaneous pre-trough activations per nurse (n_s) within a 12-hour session.

To test whether this heterogeneity exceeded random expectations, we compared the observed distribution with a permutation-based null model that randomly redistributed activations among nurses while preserving both the number of nurses and the total number of events.

In more than three quarters of the sessions (237/311), the observed inequality—quantified using the Gini coefficient—exceeded the null expectation. This difference was statistically significant in 92 of 311 sessions (p ≤ 0.05; Fig. R2a–b). These results indicate that the observed heterogeneity reflects genuine biological variation in pacemaking propensity among nurses, with some individuals contributing more frequently than others.

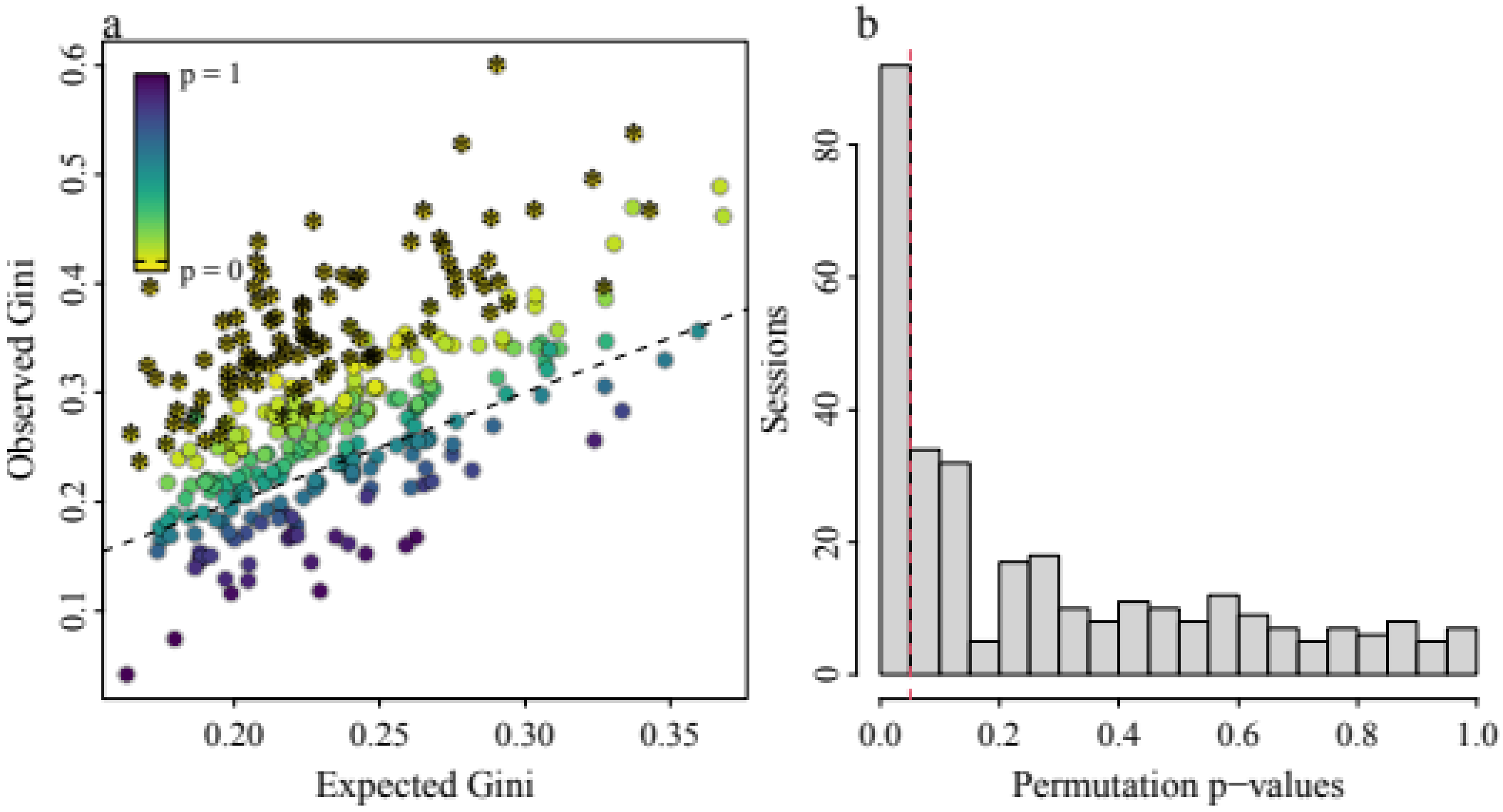

Figure R2. Pacemaking inequality relative to a permutation null model. (a) Observed Gini coefficients for the distribution of spontaneous pre-trough activations among nurses in each session compared with values expected under a null model that randomly redistributes activation events among nurses. (b) Distribution of p-values from these comparisons across sessions (dashed line: p = 0.05).

We have now incorporated the analysis of the number of spontaneously activating ants per trough and its relationship with colony size in the "Activity pacemaking" results section, as it directly addresses the reviewer's question about how pacemaking scales with colony size (lines 213-218). The additional analyses examining heterogeneity among nurses were not added to the main text for two reasons: (i) they rely on caste-specific roles that are introduced later in the Results (Fig. 6d, Fig. 7), and (ii) they involve defining worker categories using thresholds on social maturity values, which, as Reviewer 2 noted, can be somewhat arbitrary and should therefore be handled with care. Including these analyses at this stage would both disrupt the logical flow of the Results and risk over-emphasising threshold-based classifications; however, we would be happy to include them if the editor feels they would substantially strengthen the manuscript.

- what do the trajectories of pacemaker ants look like within the nest? do they wander around until they bump into their neighbors? i understand there is no evidence for negative feedback when they contact a stationary neighbor: do the pacemakers just run around for the duration of activity cycle? or do they stop early?

Thank you for this interesting question. We agree that examining the detailed trajectories of pacemaker ants following spontaneous activation would be very informative. However, addressing this would require a substantial new analysis of individual movement trajectories after activation events. As the first author who

developed the analytical pipeline has since left academia, we are unfortunately not in a position to undertake this additional analysis for the present revision.

- how is the number of contacts between ants defined? how sensitive are your results to error in this classification?

Physical contacts were inferred using a geometrical approach in which each ant was represented by a trapezoid aligned with its body axis and extending from the antennae tips to the end of the gaster. A contact was recorded when the front end of one ant's trapezoid fell within that of another. This procedure is now described in the Methods section (lines 403-405).

This contact inference method has been validated in previous studies and shown to be robust to minor tracking errors (e.g., Mersch et al. 2013; Stroeymeyt et al., 2018; Richardson et al., 2022).

- does the 'hitting speed' of an individual switching ant correlate with the activation probability of its (individual) target? this seems important to support the claim in Fig. 7c,d.

We thank the reviewer for this suggestion. To address this, we analysed physical encounters in which an active switching ant contacted an inactive receiver ant. We quantified the association between hitting speed (the mean speed of the active ant during the 10 seconds preceding contact) and the probability that the receiver became active within the subsequent 10 seconds.

Using a mixed-effects generalised linear model (GLMM), we found a significant positive association between hitting speed and receiver activation probability. We also detected a significant interaction between hitting speed and nest architecture (χ^2= 108.2, $p < 0.0001$), indicating that receivers in double-chamber nests exhibited higher sensitivity to high-speed impacts than those in single-chamber nests. Specifically, a 1 body-length.s^{-1} increase in hitting speed corresponded to a 6.43-fold increase in the odds of activation in double-chamber nests compared with a 4.29-fold increase in single-chamber nests.

We have incorporated these results into the main text (lines 288-295) and provide methodological details and a visualisation in the Supplementary Information (Fig. S17).

- some of the language (eg. line 217) implies that pacemaker ants actively track colony activity cycles to decide when to spontaneously activate. the analyses presented do not explicitly show this; I would edit the text for accuracy

Thank you for pointing this out. We agree that the use of 'track' and 'anticipate' could imply a level of cognitive awareness that our data do not support. To focus on the pacemakers' tendency to spontaneously switch on prior to collective activity troughs, *without* attributing intent to the ants, we have revised the text as follows (lines 231-234):

"These results suggest that by shielding the inner parts of the nest from external noise, modular architecture enhances the detectability of low-activity states. This may promote spontaneous pacemaker activations immediately prior to the activity trough, thereby initiating the next activity cycle."

- different figures use different notation that is slightly confusing: eg. I for intermediate vs I for inner.

We have revised the notation throughout the manuscript and figures to avoid ambiguity between "I" for inner and "I" for intermediates. Specifically, occurrences where inner locations were previously abbreviated as "I" have been replaced with "In." throughout the main text and figures, while the abbreviation "I" for intermediates remains unchanged.

- please check for typos and consistency: eg some panels are missing labels and significance markers, some references are formatted incorrectly (eg line 388)

Thank you. We have re-checked the manuscript and corrected a few typos and the reference error on L.388 (now L. 409). We have also added the missing panel labels and the significance markers in panel 7d. As far as we can tell, the only panels still lacking significance markers are panels 6a and 7c. These correspond to statistical models in which the interaction term was not significant, although both main effects were significant. In such cases, adding pairwise significance markers between bars could be misleading, as this would imply specific comparisons that were not tested. We have therefore not added significance markers to these panels and instead report the statistical results for the main effects in the figure legends.

Reviewer #2 (Remarks to the Author):

This manuscript presents an elegantly executed analysis of collective activity dynamics in *Leptothorax acervorum* colonies across single- and double-chamber nest architectures. The dataset is impressive in scale, the tracking quality is high, and the multi-layered analytical pipeline—combining contact networks, spectral characterization, activation dynamics, and transmission-chain inference—is sophisticated and innovative. The question of how modular nest structure affects collective oscillations of ants' activity is biologically meaningful and potentially inspiring for other systems. The core results regarding oscillatory dynamics and the spatial modulation of activity waves are compelling.

We thank the reviewer for their positive assessment of our work and for their clear summary of its scope and contributions.

However, I have several substantive concerns about the interpretation of the results—particularly regarding (i) the role of "nurses" in initiating activity cycles, and (ii) the proposed function of "intermediates" as activity transfer specialists. These concerns arise from how community affiliation, spatial structure, and task categories interact in the analytical pipeline. Addressing the points below will substantially strengthen the manuscript's conceptual clarity and generality.

1.The maturity axis and task groups depend strongly on the manual choice of community number in FacetNet. FacetNet requires the user to predefine the number of communities (K). Since the authors chose K = 2, the resulting "social maturity" score necessarily represents a projection onto two user-specified community poles. All workers must therefore lie somewhere on this single axis, regardless of whether the natural structure of the colony is bimodal, trimodal, or continuous. The subsequent definition of three behavioral groups (nurses, intermediates, foragers) emerges from thresholding of this

continuous 0–1 maturity score, rather than from FacetNet itself. The maturity distribution is bimodal, not trimodal, so the intermediate group arises from spatial gradients in contact patterns rather than from a formally detected community. Because many downstream analyses—including leadership, switching behavior, and activation patterns—use these categories, the conclusions about "distinct nurse and intermediate roles" may partially reflect the choice of K = 2 and threshold placement rather than emergent biological groupings.

Thank you for this thoughtful and important point.

As explained in our earlier reply to Reviewer 1, the choice of K = 2 was not imposed a priori. Rather, this choice was guided by the empirical structure of the contact networks: modularity was highest when the networks were partitioned into two overlapping communities (Fig. S4), and decreased when additional communities were imposed. This pattern is consistent with previous work across several ant species [53–55].

We agree that under this overlapping community framework, individuals are positioned along a continuous maturity axis reflecting their graded affiliation with the two community poles, and as the reviewer notes the resulting distribution is strongly bimodal. Importantly, however, a two-community overlapping solution naturally produces an overlap region between the two poles, comprising individuals that cannot be classified as belonging unambiguously to either community, but instead share substantial affiliation with both.

Prior spatial analyses in *L. acervorum* also support the interpretation that these individuals represent a biologically meaningful category. Richardson et al. (2023, *Nat. Commun.*) identified 3–4 worker categories using discrete community detection and found systematic behavioural differences between nurses and foragers on one hand and workers belonging to intermediate communities on the other.

The specific maturity thresholds ($m < 0.25$; $m > 0.75$) used to define intermediates were adopted from previous work [53] to maintain consistency with established definitions in this species and to facilitate interpretability. Crucially, however, our key behavioural analyses (e.g., leadership and switching behaviour; Figs. 6d and 7e) were conducted using the *continuous maturity scores* rather than the thresholded behavioural categories. Our conclusions therefore do not depend on the precise placement of these thresholds.

Consistent with this, while we agree that leadership varies monotonically with maturity (see next reply), location switching exhibits a clear unimodal relationship that peaks at intermediate maturity values (Fig. 7e). The description of intermediates as long-range connectors therefore reflects the empirical concentration of switching behaviour in the central region of the maturity continuum and is biologically meaningful.

2. Leadership varies smoothly across maturity, complicating categorical interpretations. Figure 6d shows leadership is flat from m = 0 to 0.5. Thus, a substantial number of early-intermediate individuals exhibit leadership behavior, even though the manuscript categorizes them as non-nurses. This creates a mismatch between the continuous maturity metric and the categorical interpretation that "nurses initiate activity cycles."

The authors might consider rephrasing this conclusion more broadly (e.g., “low-maturity individuals”), or analyzing initiation rates as a function of continuous maturity.

Thank you for this helpful suggestion. We agree that leadership varies continuously with maturity and that categorical terminology may obscure this graded pattern. We have therefore rephrased the relevant text (L. 257) to refer to “low-maturity individuals” rather than strictly to “nurses,” and have made corresponding adjustments throughout the manuscript to ensure consistency.

3: Spatial adjacency may underlie the observed “messenger” role of intermediates. Many of the observed “messenger-like” behaviors (high switching frequency, bridging inner and outer regions) appear consistent with intermediates' spatial positions—they occupy the region between the nurse- and forager-dominated zones. In any spatially structured excitable system, units located between two dense compartments will naturally serve as conduits for propagation. This suggests that intermediates' apparent transfer role may arise from topology rather than specialized task differentiation. This pattern is conceptually similar to how Giant honeybees spread shimmering vibration waves across the nest.

Thank you for this important point. We agree that the most parsimonious explanation for intermediates acting as activity conduits lies in their spatial and topological position between the nurse- and forager-dominated regions of the nest. **Individuals with intermediate maturity values occupy this interface and switch between locations most frequently (Fig. 7e), naturally allowing them to transmit activity between compartments. We have clarified the Discussion to emphasise this structural interpretation and to avoid implying that intermediates constitute a specialised task group (lines 355-359). We also added a brief comparison with the shimmering defence waves of giant honeybees (*Apis dorsata*), where signal propagation spreads across the nest surface through local transmission between neighbouring individuals (lines 360-362).**

4: The definition of “activity phases” (low/high) need to be clarified. The four activation-phase categories (low–rising, low–falling, high–rising, high–falling) play an important role in interpreting spontaneous activations. While the slope definition is clear, the threshold separating "low" from "high" activity is not explicitly defined in the Methods. This makes it difficult to assess or reproduce the phase-specific findings in Figure 5.

Thank you for pointing this out.

The generalized additive models underlying Fig. 5 were fit using the raw, continuous values of instantaneous activity level and activity slope. The analysis therefore did not involve thresholding or categorical phase definitions at any stage, which is why no threshold value was mentioned in the Methods.

The four phase labels (low–rising, low–falling, high–rising, high–falling) were overlaid on the resulting two-dimensional response surfaces only to facilitate intuitive interpretation, but were not used in the statistical analysis.

For visualisation purposes only, the separation between “low” and “high” activity was defined using a threshold of 0.5 on the proportion of active ants. This threshold is indicated by the horizontal dashed lines in Figures 5b–j. We have now clarified this in the figure caption.

5: Potential circularity between spatial structure and inferred behavioral roles was not addressed. Community affiliation, maturity, inner/outer zoning, leadership, switching rates, and propagation patterns all derive from the same spatially structured trajectory data, there is a possibility of circular inference:

1. Spatial layout determines contact network structure
2. Contact network determines "maturity"
3. Maturity determines task labels (nurse/intermediate/forager)
4. Spatial measures then correlate with these labels
5. Conclusions treat these labels as independent Behavioral roles

We appreciate the reviewer's concern regarding potential circularity arising from the shared spatial basis of network structure, maturity, and behavioural measures. Importantly, the main conclusions of the study—concerning how nest architecture modulates activity transmission and coupling between spatial compartments—do not depend on the precise definition of behavioural roles or task labels.

Nevertheless, we were aware of this potential circularity in some of our analyses. To address this, we explicitly tested whether our leadership metric, ∆k, could be explained purely by spatial location. As described in L. 260-265 and Fig. S16, we repeated the activity transmission analysis using only within-zone chains (inner-to-inner and outer-to-outer), thereby controlling for spatial compartment. We found that the negative relationship between social maturity and leadership persisted even when transmission chains are restricted to a single spatial zone. In other words, even when located in the outer parts of the nest, nurse-like workers are more influential than forager-like workers, and even when located in the inner parts of the nest, forager-like workers are less influential than nurse-like workers. This confirms that the result shown in Fig. 6d is not simply an artefact of spatial division of labour.

<u>Reviewer #3 (Remarks to the Author):</u>

In this manuscript, the authors thoroughly examine social network dynamics related to activity rhythms in nests of Leptothorax acervorum. They focus on comparing these dynamics in one-chamber vs. two-chamber artificial nests. They observe 13 colonies that are each housed in both one-chamber and two-chamber nests (for paired analysis) and are filmed continuously. Analyses include identifying the spatial location of rhythm-setting activity (inner areas), which behaviorally specialized workers tend to initiate activity (nurses), where behaviorally specialized workers tend to be located in colonies (nurses towards the inside), and how activity is coordinated among locations and chambers (e.g., less coupled in multi-chamber nests). The primary method used is automated tracking of individuals and classification of social interactions. I appreciate the pairing of manipulative/artificial aspects (namely the nest architecture) with fairly non-invasive monitoring of natural behavioral dynamics in this species. The authors identify the broader significance of this study as relating to oscillations and coordination between different oscillating modules, recurring phenomena in nature.

I find this study to be thoughtful and detailed. I think the references are appropriate and I believe this study is original. I have no major complaints about the methods or interpretations, but I have a few smaller comments mainly related to improving clarity.

We thank the reviewer for their careful reading of our manuscript and for the helpful comments provided below.

Detailed comments:
Line 25: I think the em dash spacing needs to be fixed.

Thank you for spotting this. We have now corrected the spacing.

Line 29: Should this be “turn” singular? Not sure of the intended grammar.

Thank you for noting this. The typo has now been corrected.

Line 30: Consider removing “the” before METs.

We retained the definite article here because “the Major Evolutionary Transitions” refers to a specific, established conceptual framework in evolutionary biology.

Line 71: Please provide more clarity here (rather than just in the method) about the six-day time period vs. the 12-hour filming periods.

Thank you for this suggestion. We have added a brief clarification in the main text (L. 77-81) describing the relationship between the six-day housing period and the 12-hour filming intervals used in the analyses.

Line 77: From the description of the artificial nests, it seems like the top portions would be clear glass. While this light/dark regimen is fairly standard, would having ants that otherwise nest inside of acorns or rock crevices be exposed to 12 hours of direct light possibly influence behavioral rhythms?

Thank you for pointing this out. The roof of each artificial nest consisted of an IR-passing glass filter that blocks visible wavelengths while allowing infrared illumination for filming and real-time tracking. Because ants do not perceive infrared light, the nest interior remained dark to the ants throughout the light–dark cycle. While this was described in the Supplementary Information (‘Experimental Conditions’), we have now added a brief clarification in the main text to make this explicit (L. 81-82).

Line 88: Consider rephrasing for clarity; since each colony was house in both one- and two-chamber nests, does it make sense to phrase it as “these nests contained more fully specialized workers” when they are the same workers as before? Maybe you mean the ants acted in more specialized ways in this environment.

Thank you for this suggestion. We have rephrased the sentence to clarify that the effect concerns behavioural polarisation rather than the identities of the workers: “... suggesting that modular nest architecture induces a stronger polarisation of workers towards the full nurse and forager profiles.”

Line 95: Please specify the grid size here.

Thank you for noting this. We have now specified the grid resolution (grid width = ½ worker body length; L. 105-106).

Line 100: How was the grid done for two-chamber nests to allow for comparison between the two chambers?

In both nest geometries, the same grid resolution (grid width = ½ worker body length) was used, and the grid was overlaid across the entire nest footprint. As both nest geometries had the same total area, this allowed spatial comparisons to be made at equivalent spatial resolution within and between chambers. We have clarified this in the revised Methods section (L. 437-440).

Line 169: I would mention your threshold for determining inactivity here, not just in the methods.

Thank you for this suggestion. Because overall activity levels varied among colonies, we did not apply a single fixed threshold to define inactivity. Instead, we used the multivariate approach described in Richardson et al. (2021, 2022). We agree that this procedure was not described clearly enough in the original submission. We have therefore expanded the description in the main text (L. 180-182) and revised the Methods section to more clearly direct readers to the full explanation in the Supplementary Information (L. 408-412).

Line 253: Please clarify why the choice was made to focus on times when the second location was quiescent prior to switcher arrival.

Thank you for this helpful comment. We have clarified that we focused on cases in which the second location was quiescent prior to switcher arrival in order to maximise our ability to detect genuine activity transmission events, rather than conflating them with ongoing activity. We have added this explanation to the revised manuscript (L. 269).

Line 282: Somewhere in the discussion I would really like to see information on the relationship of your results to real Leptothorax acervorum biology/nests. Given that they live in pre-existing structures that seem to typically have only one large interior space, do they ever take steps to subdivide them into chambers?

Thank you for this thoughtful question. *Leptothorax* ants typically nest in pre-existing cavities such as acorns, rock crevices, or decaying wood. Although these nest sites are often described as single internal spaces, structural data (e.g., CAT scans of acorn nests in Varoudis et al., 2018) show that they can exhibit substantial internal complexity, including folds and invaginations that create partial spatial subdivision. Nests in decaying wood also rarely consist in simple single chambers; instead, they often consist of multiple cavities connected by a network of galleries.

To clarify the ecological context of our experimental manipulation, we have expanded the Introduction to briefly discuss the natural variability of nest architecture in *L. acervorum* (L. 56-62).

Line 387: Fix this reference.

Thank you for noting this. We have now corrected the reference.

Line 514: Is the R code also available somewhere?

The analysis code and raw data have been uploaded to a Zenodo repository (https://doi.org/10.5281/zenodo.15020312). The repository contains the data files and the fully annotated R scripts used for the analyses, together with documentation of the software requirements. The link is now provided in the Data and Code Availability section of the main text. The repository is currently under embargo and will be made fully open access upon publication of the manuscript.

In this manuscript, the authors thoroughly examine social network dynamics related to activity rhythms in nests of *Leptothorax acervorum*. They focus on comparing these dynamics in one-chamber vs. two-chamber artificial nests. They observe 13 colonies that are each housed in both one-chamber and two-chamber nests (for paired analysis) and are filmed continuously. Analyses include identifying the spatial location of rhythm-setting activity (inner areas), which behaviorally specialized workers tend to initiate activity (nurses), where behaviorally specialized workers tend to be located in colonies (nurses towards the inside), and how activity is coordinated among locations and chambers (e.g., less coupled in multi-chamber nests). The primary method used is automated tracking of individuals and classification of social interactions. I appreciate the pairing of manipulative/artificial aspects (namely the nest architecture) with fairly non-invasive monitoring of natural behavioral dynamics in this species. The authors identify the broader significance of this study as relating to oscillations and coordination between different oscillating modules, recurring phenomena in nature.

I find this study to be thoughtful and detailed. I think the references are appropriate and I believe this study is original. I have no major complaints about the methods or interpretations, but I have a few smaller comments mainly related to improving clarity.

Detailed comments:

Line 25: I think the em dash spacing needs to be fixed.

Line 29: Should this be “turn” singular? Not sure of the intended grammar.

Line 30: Consider removing “the” before METs.

Line 71: Please provide more clarity here (rather than just in the method) about the six-day time period vs. the 12-hour filming periods.

Line 77: From the description of the artificial nests, it seems like the top portions would be clear glass. While this light/dark regimen is fairly standard, would having ants that otherwise nest inside of acorns or rock crevices be exposed to 12 hours of direct light possibly influence behavioral rhythms?

Line 88: Consider rephrasing for clarity; since each colony was house in both one- and two-chamber nests, does it make sense to phrase it as “these nests contained more fully specialized workers” when they are the same workers as before? Maybe you mean the ants acted in more specialized ways in this environment.

Line 95: Please specify the grid size here.

Line 100: How was the grid done for two-chamber nests to allow for comparison between the two chambers?

Line 169: I would mention your threshold for determining inactivity here, not just in the methods.

Line 253: Please clarify why the choice was made to focus on times when the second location was quiescent prior to switcher arrival.

Line 282: Somewhere in the discussion I would really like to see information on the relationship of your results to real *Leptothorax acervorum* biology/nests. Given that they live in pre-existing structures that seem to typically have only one large interior space, do they ever take steps to subdivide them into chambers?

Line 387: Fix this reference.

Line 514: Is the R code also available somewhere?
