# Supplementary material for: Modular nest structure influences activity cycle synchronisation in ant colonies
Source: Commun Biol. 2026 Jun 27;9:1317. doi: 10.1038/s42003-026-10560-x (PMC13649663; doi:10.1038/s42003-026-10560-x)
Supplement: Supplementary file 2 [file 42003_2026_10560_MOESM2_ESM.pdf]

Supporting Information: Modular nest structure influences activity cycle synchronisation in ant colonies

Thomas O. Richardson[1,2], Matthias Rüegg[2,3], Zoe Pritchard[4,5], Laurent Keller[6], and Nathalie Stroeymeyt[1,2]

[1]*School of Biological Sciences, University of Bristol, Bristol, UK*
[2]*Department of Ecology and Evolution, University of Lausanne, Lausanne, Switzerland*
[3]*Institute of Mechanical Engineering, EPFL, Lausanne, Switzerland*
[4]*Bozeman Field School, 2090 Stadium Drive, Bozeman, Montana, USA*
[5]*Department of Ecology Evolution and Organismal Biology, Iowa State University, Ames, Iowa, USA*
[6]*Social Evolution Unit, Cornuit 8, BP 855, 1885 Chesières, Switzerland*

Replicate	Nest architecture	Colony	Total ants	Total brood	Total trajectory fixes	Total ant time (d:h:m)	Total contacts
1	D	8	43	130	22 175 313	128:8:0	294 455
1	D	11	104	174	63 270 051	366:3:24	671 925
1	D	13	55	153	33 203 450	192:3:42	341 900
1	D	19	37	48	26 183 541	151:12:30	164 979
1	D	21	70	99	26 124 404	151:4:30	273 385
1	D	25	58	164	25 364 873	146:18:48	242 798
1	S	1	45	134	24 508 698	141:20:12	253 131
1	S	10	37	82	21 840 998	126:9:30	133 258
1	S	12	53	132	29 278 638	169:10:42	248 382
1	S	14	60	137	43 179 577	249:21:18	492 136
1	S	18	35	71	20 412 091	118:3:0	123 927
1	S	23	64	214	15 325 072	88:16:30	164 425
1	S	29	78	144	50 955 411	294:21:6	589 046
2	D	1	40	137	20 004 568	115:18:24	221 883
2	D	10	36	73	22 252 910	128:18:48	183 820
2	D	12	53	129	25 006 470	144:17:6	200 458
2	D	14	56	138	34 160 570	197:16:30	355 312
2	D	18	33	71	19 761 489	114:8:42	110 392
2	D	23	49	182	10 212 001	59:2:12	75 568
2	D	29	80	147	48 137 959	278:13:48	639 662
2	S	8	36	131	17 825 855	103:3:54	231 765
2	S	11	89	180	45 568 120	263:16:48	272 163
2	S	13	49	128	24 375 787	141:1:30	183 389
2	S	19	33	48	24 362 654	140:23:48	109 584
2	S	21	68	107	29 883 363	172:22:24	298 834
2	S	25	49	165	18 774 520	108:15:36	174 980
				Σ	742 148 383	4294:20:42	7 051 557

Table S1: **Dataset summary.** S - single-chamber nest, D - double-chamber nest. Note, the *Total ant time* column is an alternative representation of *Total trajectory fixes*, that is, the total number of barcode tag positions obtained at a frame-rate of 2 frames/second, expressed in days, hours and minutes. The *Total brood* column includes brood of all developmental stages.

**Response**	**Fixed effect**	$\beta\pm$ S.E.	**Odds ratio**	**z**	**p**
Activation	Intercept	-3.67±0.118	0.0255	-31.1	<0.0001
	N prev contacts w/ actives	0.912±0.00523	2.49	174	<0.0001
	N prev contacts w/ inactives	0.22±0.0119	1.25	18.5	<0.0001
Deactivation	Intercept	-3.05±0.065	0.0474	-46.9	<0.0001
	N prev contacts w/ actives	-0.118±0.00296	0.889	-39.8	<0.0001
	N prev contacts w/ inactives	0.00214±0.0057	1	0.376	0.707

Table S2: **Activation is contagious.** The table presents the results of two generalised linear mixed-effects models (GLMM). The first (second) model tested whether the number of contacts that an inactive (active) nestmate accumulated with active and inactive nestmates during a 10-second window influenced whether it became active (inactive) during the next 10 seconds. As in both models the response was binary (0; no state change, 1; switch), we used a binomial error structure and a logit link function. Colony identity, 12-hour session number and day/night were coded as random effects. The model-estimated coefficients (and the associated standard errors) are given in the β column. For ease of interpretation, these coefficients are also expressed as odds ratios.

Model	Main effect(s)	d.f.	AIC	Δ AIC	Rank
4	*te*(activity, slope)	879	961222	0	1
3	*s*(activity) + s(slope)	801	961376	154.3	2
1	*s*(activity)	777	961757	535.8	3
2	*s*(slope)	754	988126	26904.6	4

Table S3: **Comparing semi-parametric models predicting the dependence of IoS upon the current activity level and activity slope.** All four models were identical in all respects other than whether one or both measures of collective activity were included, and whether they were smoothed independently, or in combination. '*s*()' indicates a spline smoother, modelling the effect of activity or activity slope upon IoS. '*te*()' indicates a tensor smoother, modelling the combined effects of activity and activity slope.

**A. parametric terms**	**Coefficients, β**	**S.E.**	**t**	**p**
Intercept	0.55	0.18	3.05	< 0.0001
Location (inner - outer)	-0.28	0.21	-1.34	0.18
architecture (double - single)	0.42	0.27	1.56	0.12
Location $\times$ architecture	-0.73	0.33	-2.20	0.03
**B. smooth terms**	**Effective d.f.**	**Residual d.f.**	**F**	**p**
te(Activity, Slope) single-chamber, inner	25.7	35.6	3170	< 0.0001
te(Activity, Slope) single-chamber, outer	29.8	41.3	7450	< 0.0001
te(Activity, Slope) double-chamber, inner	44.9	62.1	4726	< 0.0001
te(Activity, Slope) double-chamber, outer	55.4	76.3	11546	< 0.0001
Colony ID	11.7	12.0	319075	< 0.0001
Ant ID	690.9	768.0	41118	< 0.0001
Session Number	11.8	12.0	1736	< 0.0001
Day or Night	0.0	1.0	0.03	0.39

Table S4: **Semi-parametric modelling for the association between activation spontaneity (IoS) and collective activity.** As the dependent variable (IoS) is a proportion, the model used a binomial error distribution, and hence the β coefficients and standard errors give the log-odds. The first four smooth terms summarise the combined effects of colony activity and activity slope on the IoS. One factor-smooth term was included for each of the four combinations of nest architecture and transition location. The last four rows summarise the random effects.

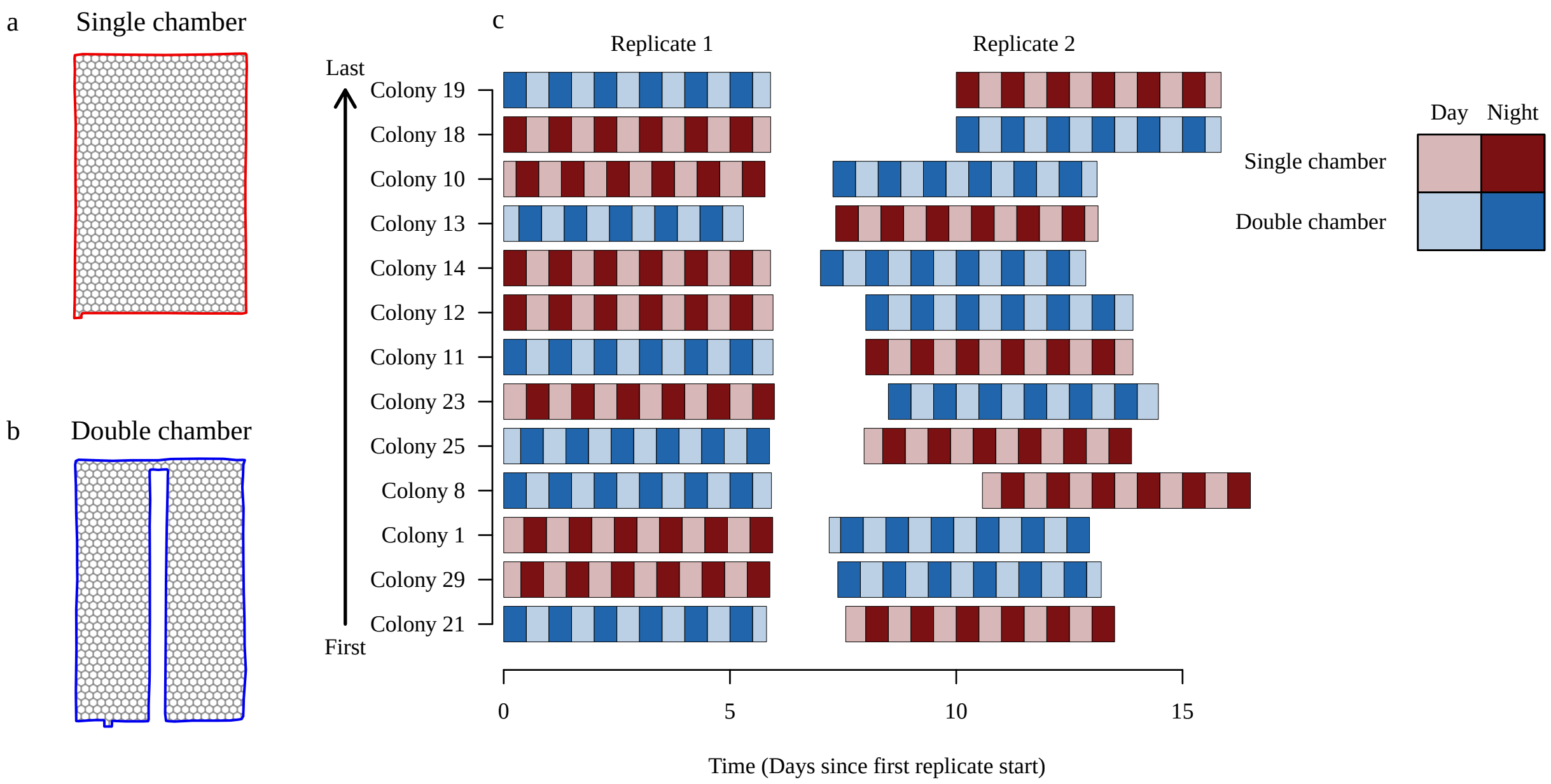

Figure S1: **Experimental schematic.** **(a-b)** Nest geometries. Both geometries have the same total area. The entrance is at the bottom-left of each nest. The grey hexagons indicate the grid used to calculate the social maturity of the typical visitor to a given site, that is, the site maturity. Hexagon widths were set to 1/2 the mean worker body length. **(c)** Schematic of the double-replicate crossover timeline. Colonies ordered by start date (bottom to top).

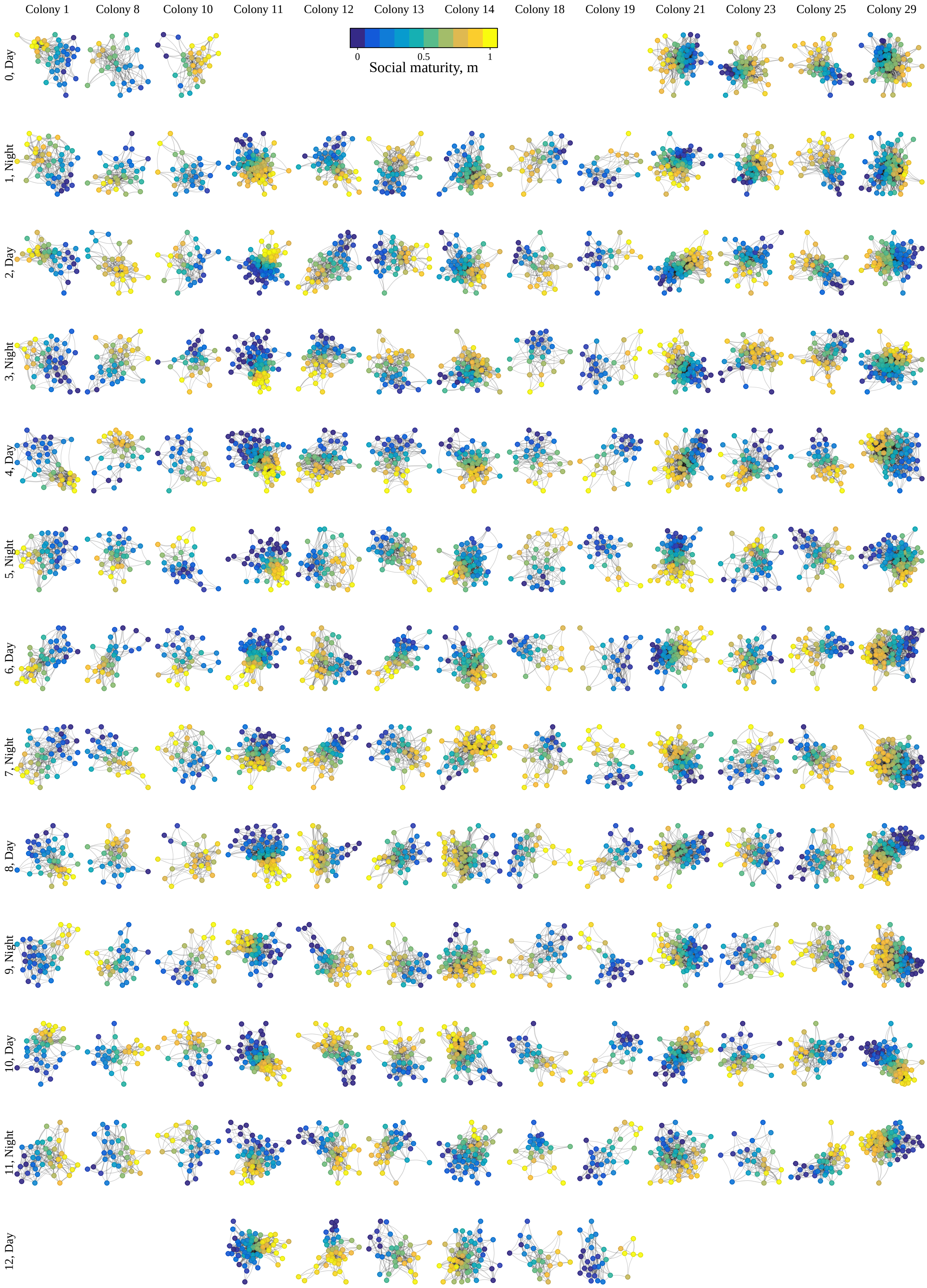

Figure S2: **12-hour contact networks for colonies in single-chamber nests.** Nodes represent individual ants, and are coloured according to the social maturity. Edges represent the number of contacts between a given pair. The network layout is determined by a force-directed algorithm which ensures that pairs that have many contacts are neighbours. To reduce distortion from peripheral nodes, nodes with degree of two or less are excluded, and only the edges with weights in the top quartile are drawn.

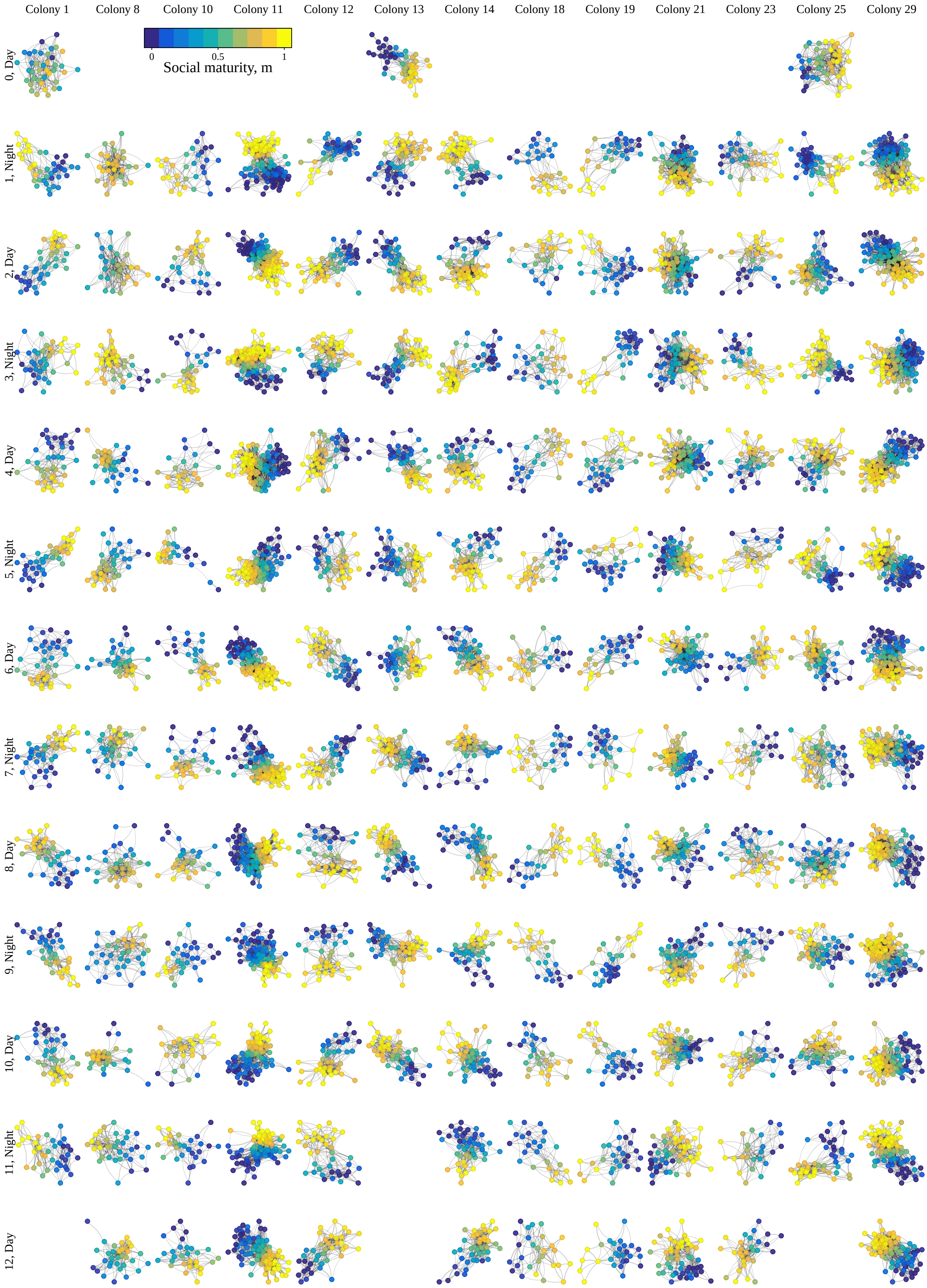

Figure S3: **12-hour contact networks for colonies in double-chamber nests.** Nodes represent individual ants, and are coloured according to the social maturity. Edges represent the number of contacts between a given pair. The network layout is determined by a force-directed algorithm which ensures that pairs that have many contacts are neighbours. To reduce distortion from peripheral nodes, nodes with degree of two or less are excluded, and only the edges with weights in the top quartile are drawn.

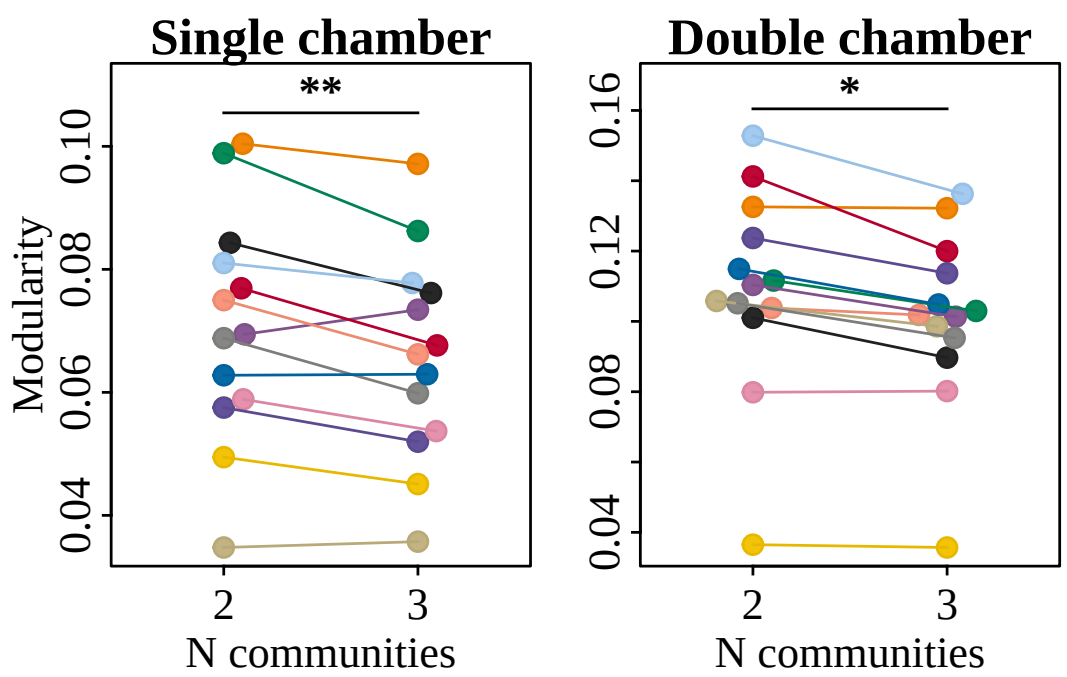

Figure S4: **Modularity is maximised when the contact networks are divided into two overlapping communities.** Points represent colony means, averaged across all 12-hour contact networks. Point colours indicate colony identities. The asterisks indicate the statistical significance for the models described in the Methods.

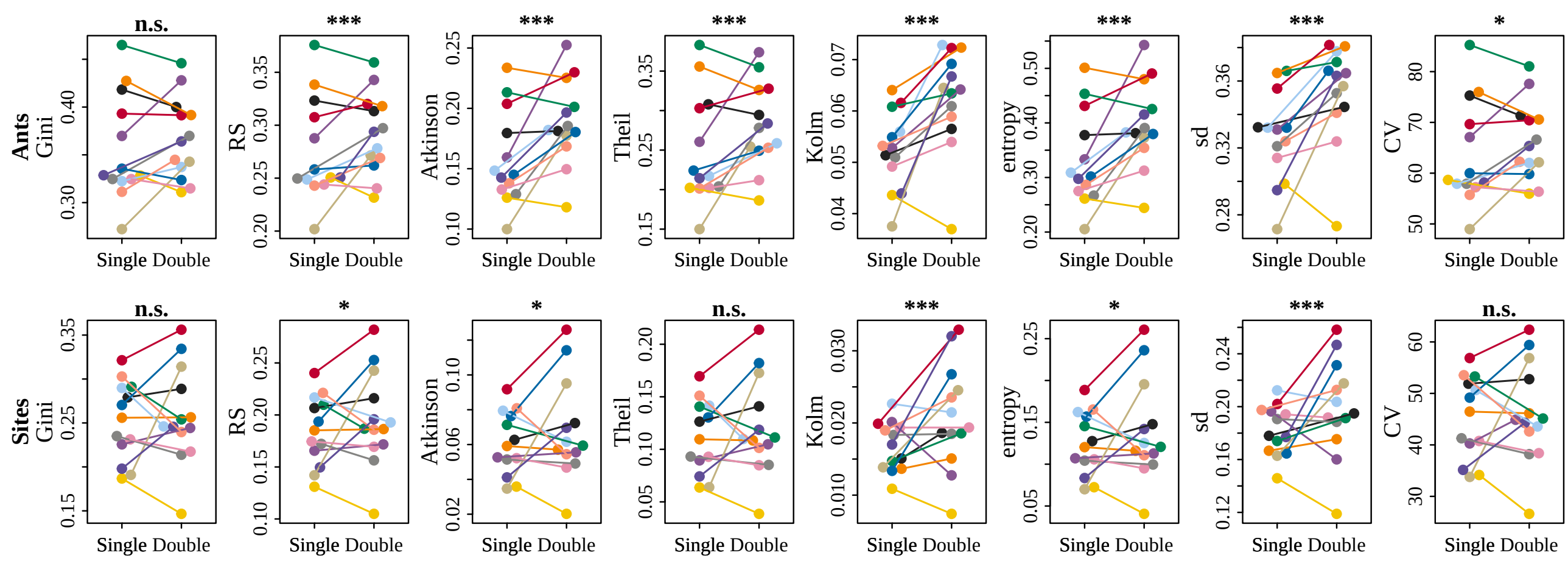

Figure S5: **Measuring heterogeneity in the distributions of social and site maturity (upper and lower rows, respectively).** Distribution heterogeneity was assessed using eight measures: the Gini coefficient (Gini), the Ricci-Schutz coefficient (RS), the Atkinson Index (Atkinson), the Theil Index (Theil), the Kolm Index (Kolm), Shannon's Entropy (entropy), the Standard Deviation (sd) and the Coefficient of Variation (CV). Points represent colony means. Point colours indicate colony identities. n.s.: p>0.05; *: $p \leq 0.05$; ***: p<0.0001 (LMM, HeterogeneityIndex $\sim$ architecture with colony and session number as random factors).

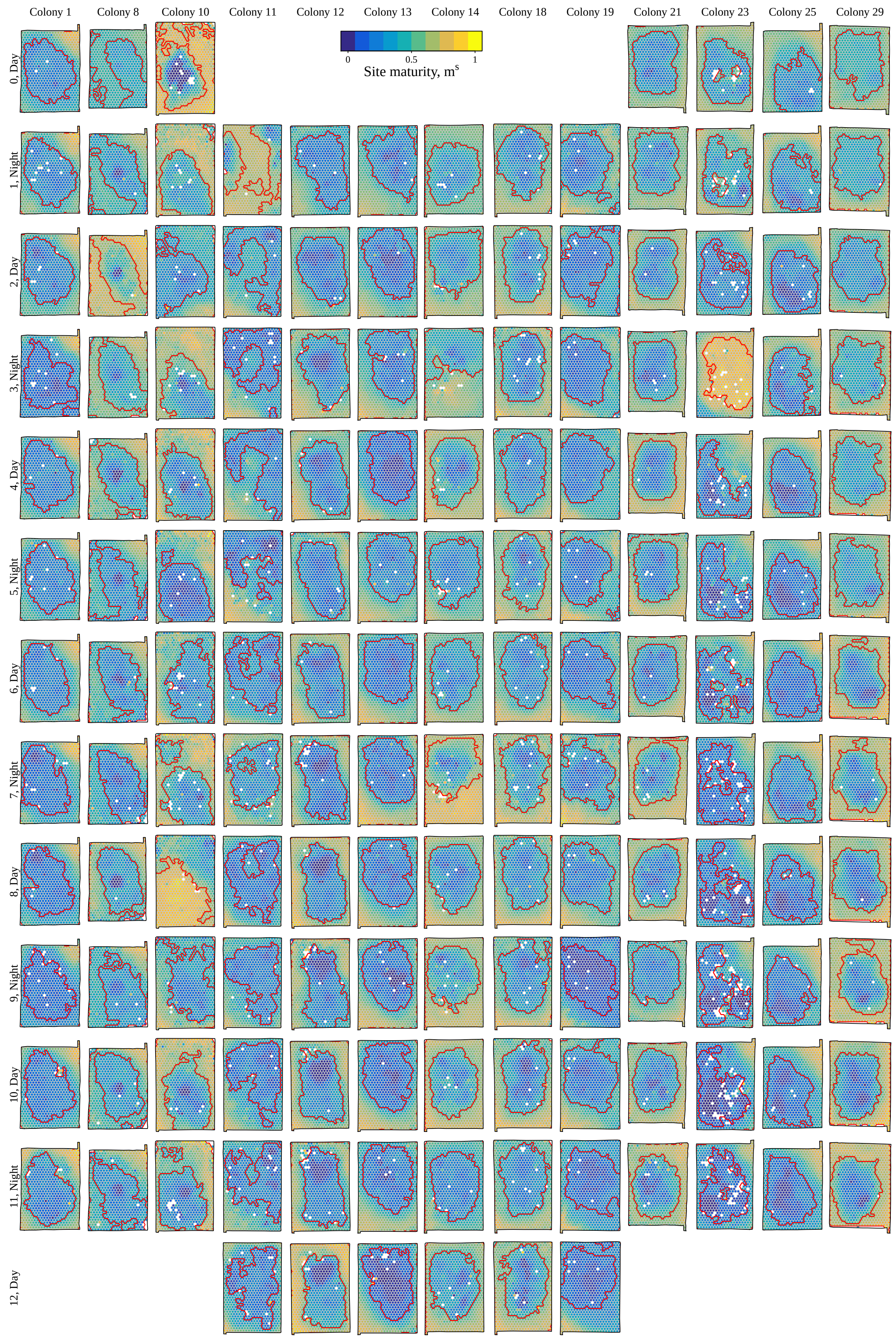

Figure S6: **Using site maturity to identify inner and outer spatial zones for colonies housed in single-chamber nests.** The red line indicates the boundary between the inner zone (mainly sites with low site maturity scores), and the outer zone (sites with high site maturity scores).

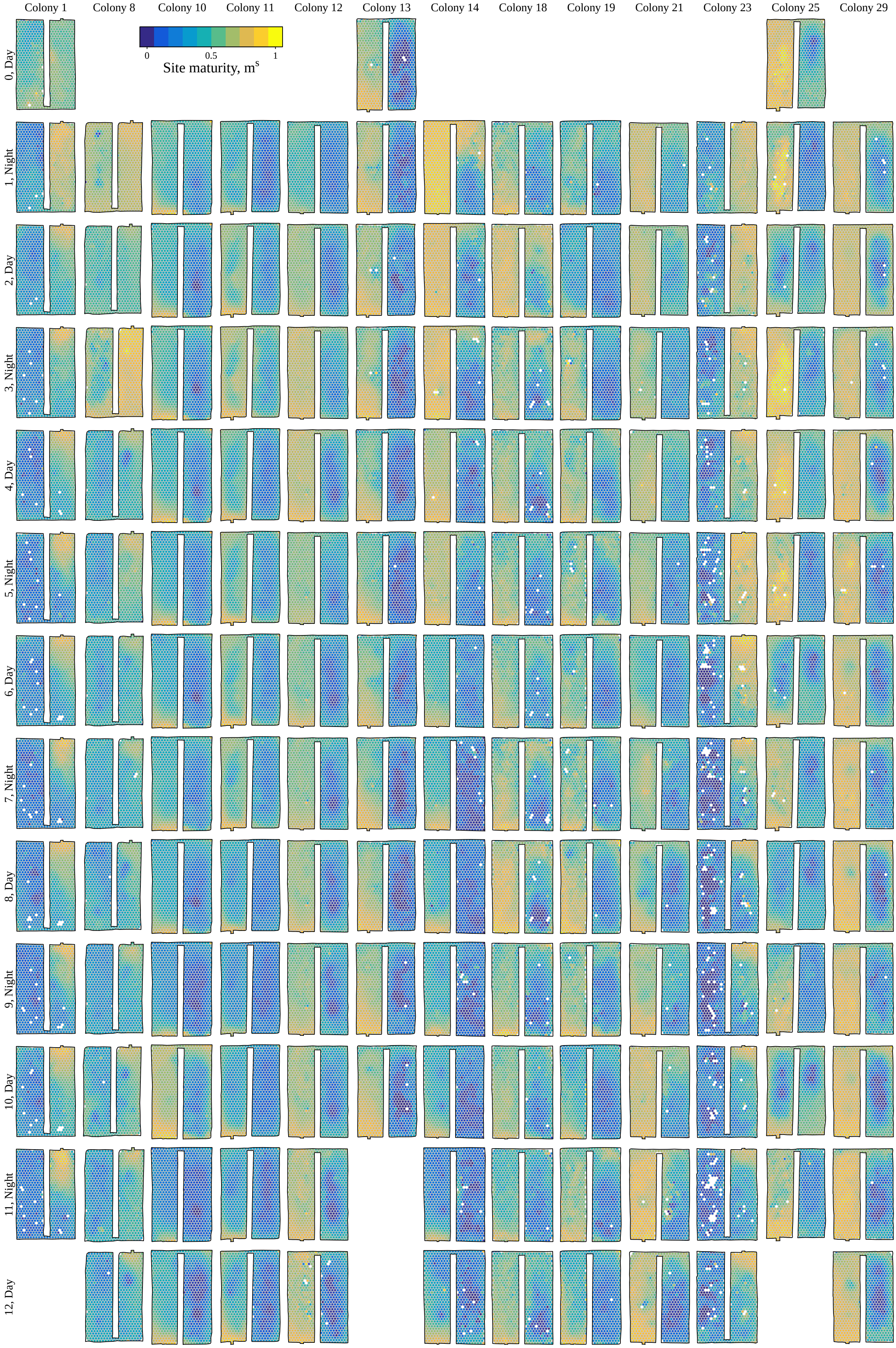

Figure S7: **Site maturity maps for colonies housed in double-chamber nests.**

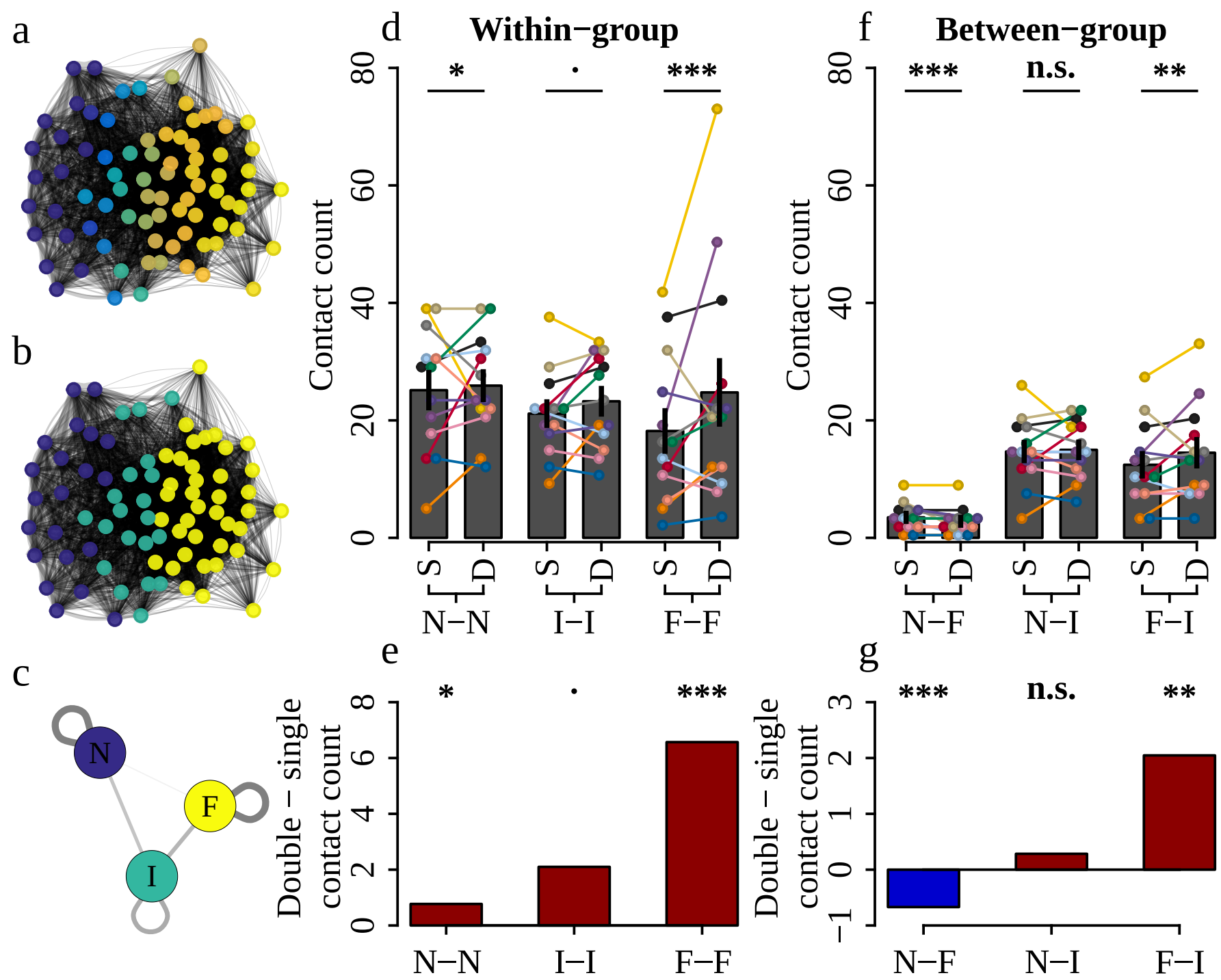

Figure S8: **Modular nest architecture modifies within-group and between-group connectivity.** Panels **(a-c)** illustrate the procedure for measuring the connection strengths within and between the two communities. **(a)** An example 12-hour contact network (Colony 29, double-chamber nest, day 8). Node colours indicate the social maturity, m. Edge shading is proportional to the total number of contacts between each pair of ants. **(b)** The 12-hour contact network with discretised task labels. Nurse specialists (those with $m < 0.25$) were assigned a 'N' label (blue), forager specialists (those with $m > 0.75$) were assigned a 'F' label (yellow), and all other workers were assigned an 'I' (Intermediate) label (green). **(c)** Coarse-grained representation of the contact network. Curved edges represent within-group connection strengths. Edge shading is proportional to the number of contacts across all pairs. **(d,f)** Median number of contacts across all within- **(d)** or between-group **(f)** ant pairs, conditioned on the nest architecture (S: single-chamber, D: double-chamber). Points show colony means. Bars and whiskers show grand means and standard errors. **(e)** Between-architecture contact number differences. Bars show the mean pairwise differences between the data points shown in panel **d**. Asterisks indicate Benjamini-Hochberg corrected post-hoc contrasts (n.s.: p>0.05, .: p<0.1, *: p<0.05, **:p<0.001, ***:p<0.0001). LMM, $\log_{10}$count $\sim$ architecture $\times$ edge type, Within-group connectivity, interaction edge:architecture, d.f.=2, χ^2=10.57, p=0.0051; Between-group connectivity, interaction edge:architecture, d.f.=2, χ^2=39.92, p<0.0001; post-hoc contrasts, double-chamber minus single-chamber, N-N: z=2.14 , p=0.049; I-I: z=1.66 , p<0.096; F-F: z=5.86, p<0.0001; N-F: z=-5.01, p<0.0001; N-I: z=0.92, p=0.36; I-F: z=3.73, p<0.00029.

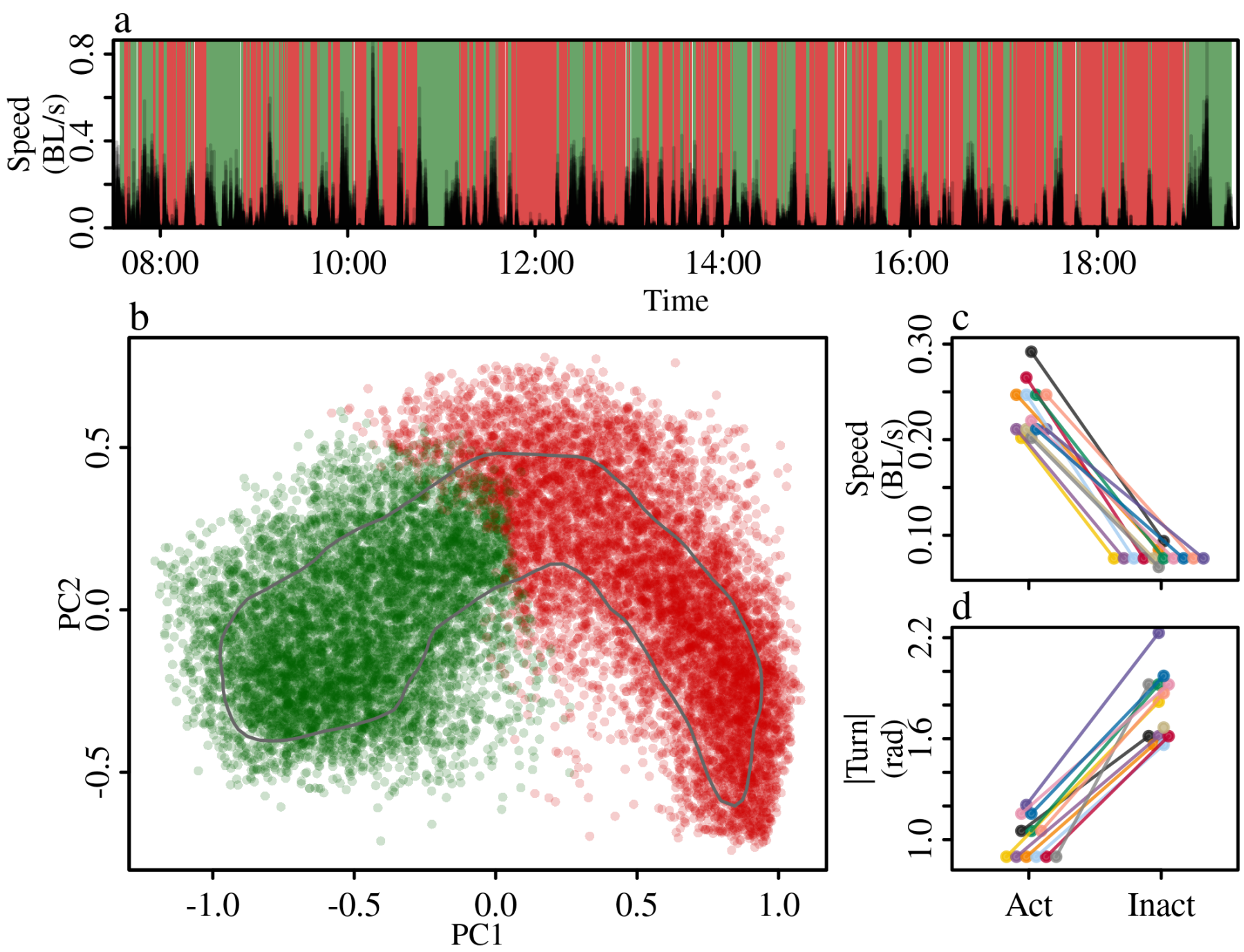

Figure S9: **(a)** Example of a 12-hour speed time-series for a single ant. Inactive bouts are coloured in red, and active bouts coloured in green. **(b)** Principal components analysis (PCA) for all trajectory segments from all ants in one colony. Each point represents a trajectory segment. Green - trajectory segments classified as active. Red - segments classified as inactive. **(c-d)** Bout speeds and turn angles, conditional upon the activity classification. Points represent colony means. Point colours indicate colony identities.

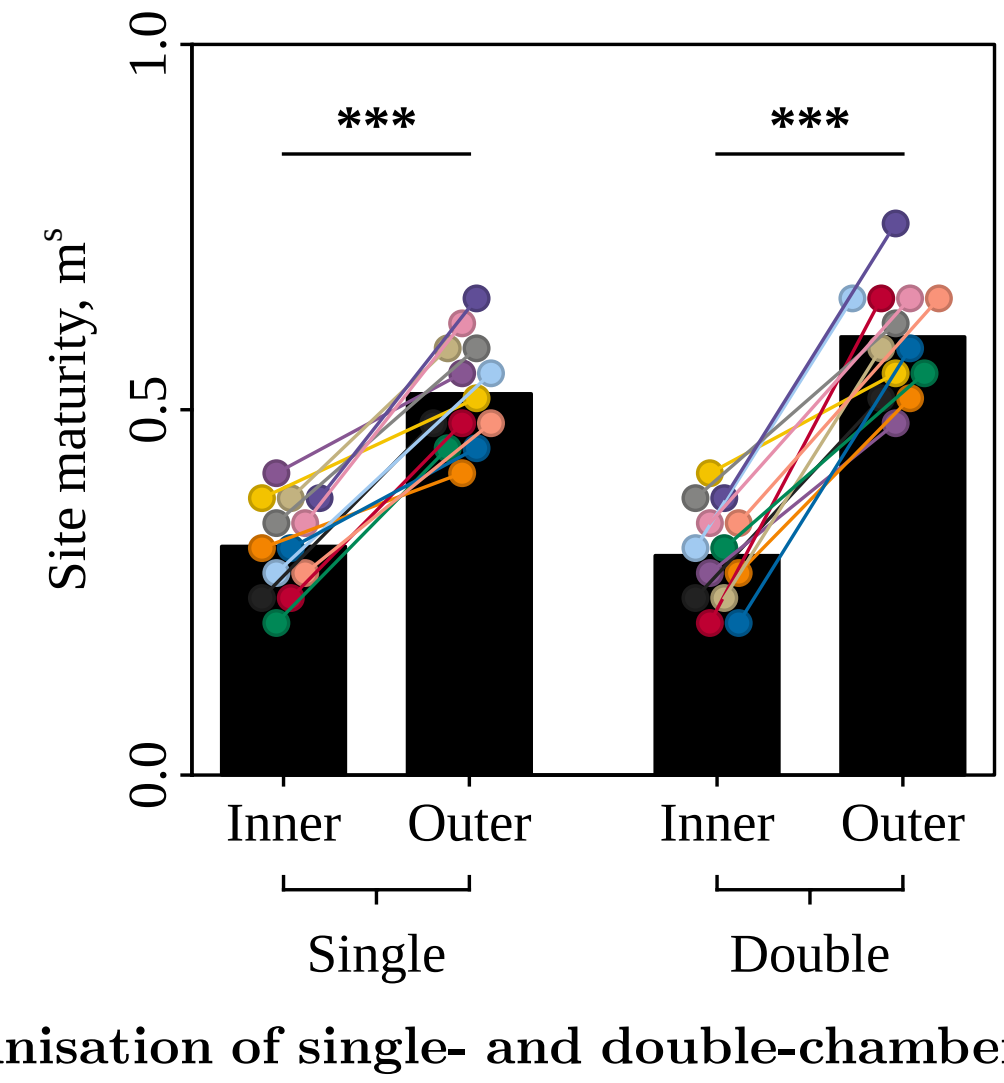

Figure S10: **Socio-spatial organisation of single- and double-chamber nests.** Site maturity of the inner and outer locations as a function of nest architecture (single-chamber nests: inner vs. outer zones; double-chamber nests: inner vs. outer chambers). Points represent colony means. Point colours indicate colony identities. Bars and whiskers show grand means and standard errors. Asterisks indicate Benjamini-Hochberg corrected post-hoc contrasts (LMM, m^s $\sim$ architecture $\times$ location, planned post-hoc contrasts, outer-inner, single-chamber nest: t=20.56 , p<0.0001; double-chamber nest: t=29.28 , p<0.0001).

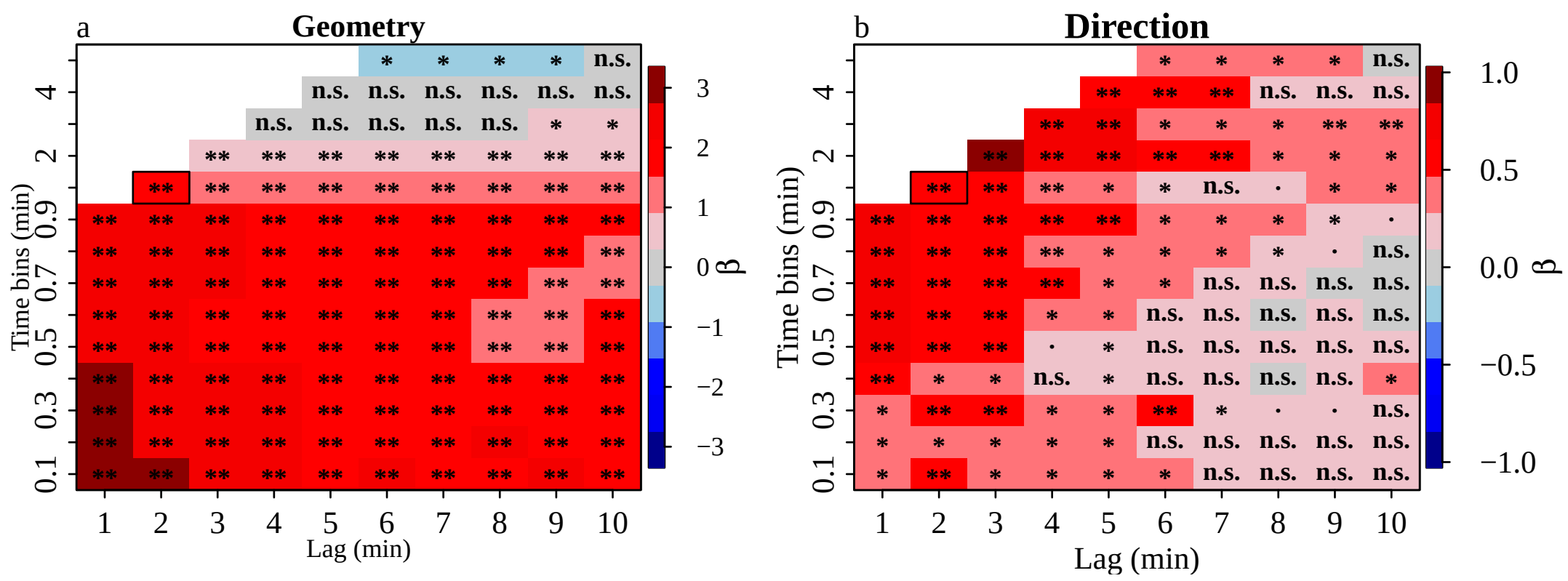

Figure S11: **Parameter sweep for Granger causality tests.** Heatmaps show the coefficients β for nest architecture (**a**) and causal direction (**b**), estimated using additive binomial GLMMs with the same random-effect structure as the model reported in the main text. Symbols in each cell indicate the statistical significance for the main effect. The cell highlighted with a rectangle indicates the parameter combination reported in the main text. Note, in the GLMM the reference category for the architecture main effect was 'double', hence positive values of β indicate that colonies housed in single-chamber nests were more likely to produce a significant Granger test result. Similarly, the reference category for the direction main effect was 'O→I', hence positive values of β indicate that significant Granger test results were more likely for the I→O direction.

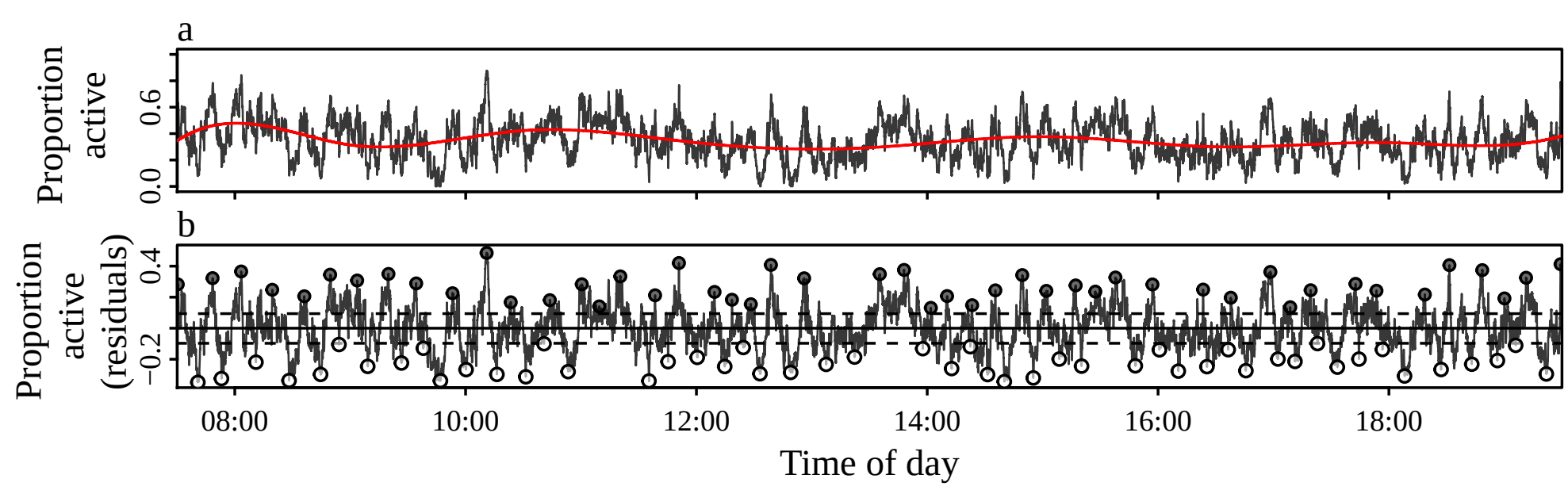

Figure S12: **Identifying local activity peaks and troughs. (a)** An example whole-colony activity time-series (colony 18, double-chamber, day 4). The red line indicates a local spline fit. **(b)** Identifying local activity peaks and troughs using the detrended activity time series, obtained from the residuals of the spline fit. Filled and empty points represent local peaks and troughs respectively. A local peak is defined when the detrended activity exceeds the 75th percentile (indicated by the upper dashed line), with the condition that successive peaks must be separated in time by at least 500 seconds.

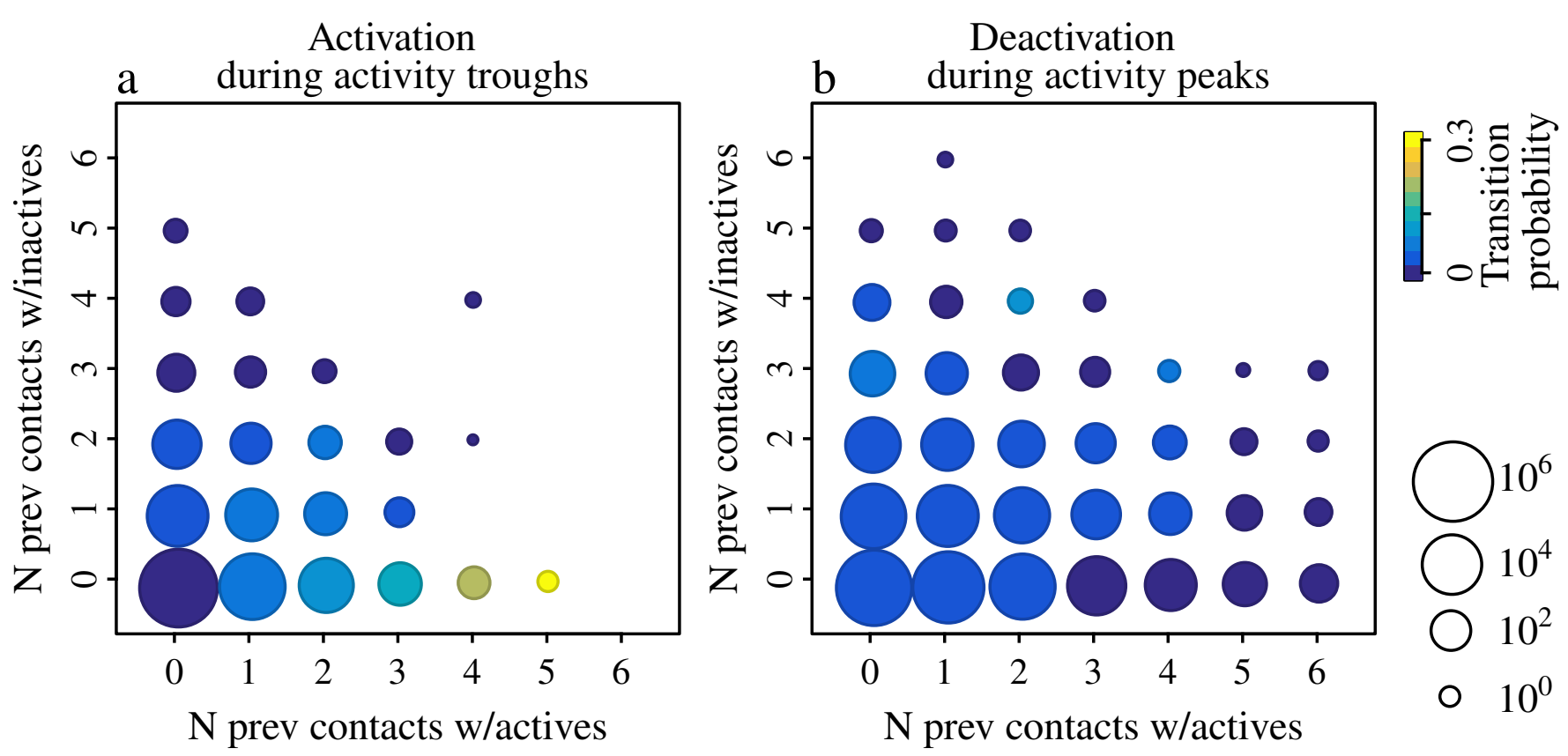

Figure S13: **Social contagion of activity but not inactivity.** Probability of an inactive ant activating in the 10 seconds following an activity trough **(a)** or of an active ant deactivating in the 10 seconds following an activity peak **(b)** as a function of the number of active (X-axis) or inactive (Y-axis) ants encountered over the 10 seconds preceding the trough/peak. Circle colour encodes the probability and circle size is proportional to the logarithm of the sample size.

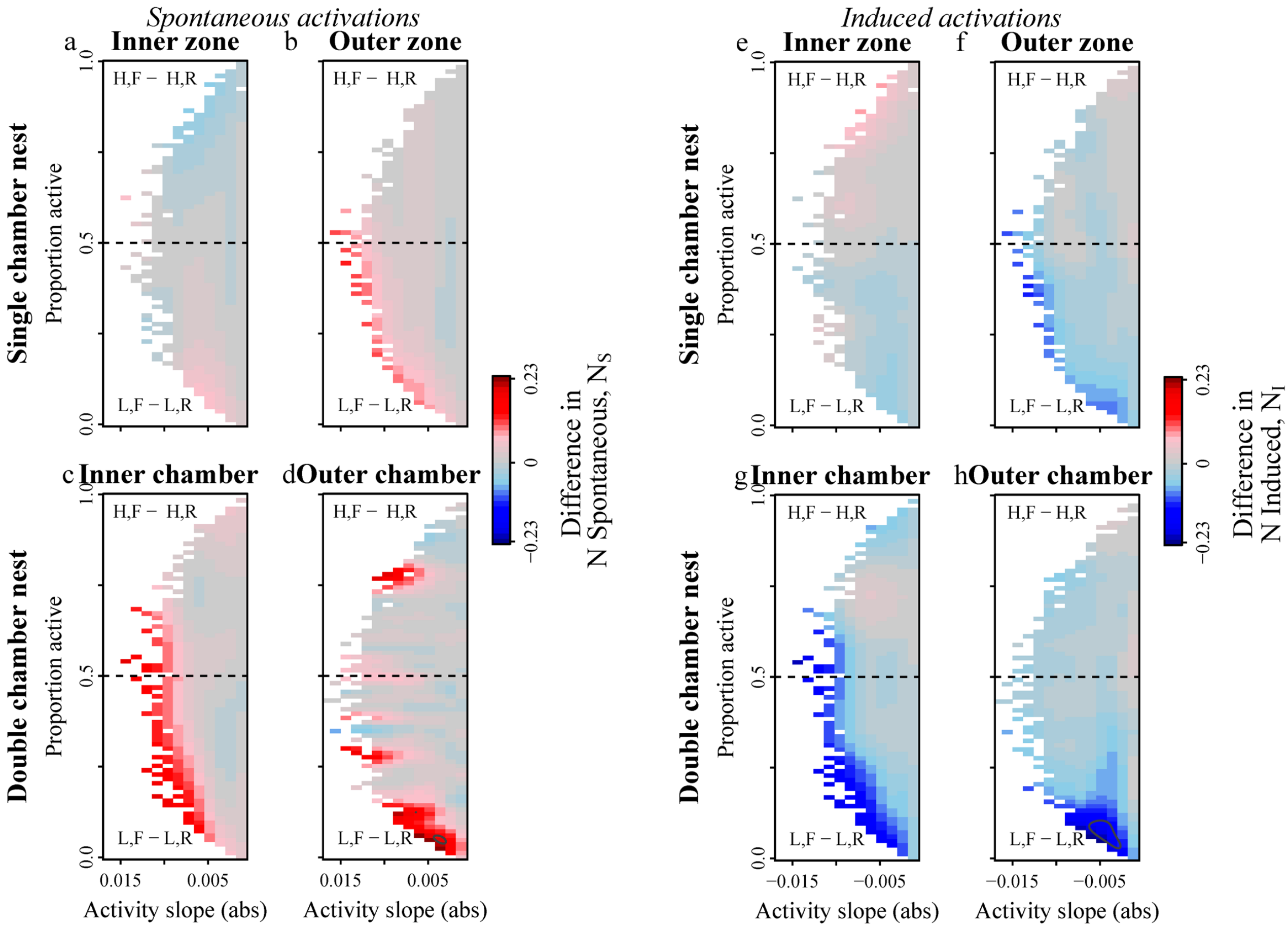

Figure S14: **Asymmetry in the total number of spontaneous and induced activations depending on the direction of activity change in the inner and outer locations of single- and double-chamber nests. (a-d)** Difference maps for total number of spontaneous activations, produced by subtracting the half of the activity map representing rising activity (positive activity slopes, right side), from the half representing falling activity (negative slopes, left side), calculated in the same way as ΔIoS in the main text. Positive values of ΔN_S indicate that falling activity is associated with more spontaneous activations than rising activity. Contours indicate areas where the N_S for falling activity significantly differs from the N_S for rising activity. **(e-h)** Difference maps for total number of induced activations, produced by subtracting the half of the activity map representing rising activity (positive activity slopes, right side), from the half representing falling activity (negative slopes, left side), calculated in the same way as ΔIoS in the main text. Negative values of ΔN_I indicate that falling activity is associated with fewer induced activations than rising activity. Contours indicate areas where the N_I for falling activity significantly differs from the N_I for rising activity.

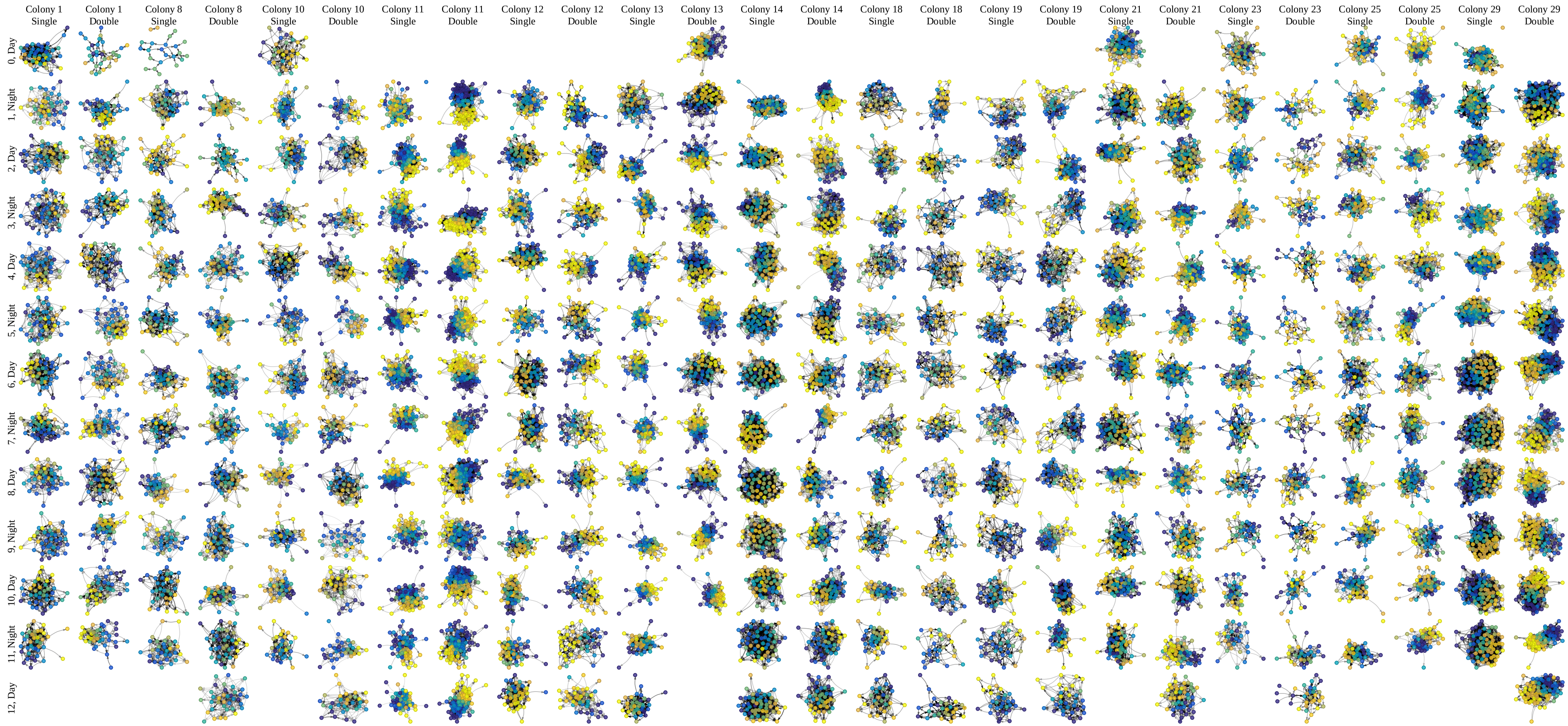

Figure S15: **Activity transmission networks.** Each network represents the aggregate transmission network for a single 12-hour session. Directed edges point from the net leader to the net follower. Edge weights indicate the absolute value of the difference between the number of times ant i led ant j, and the number of times j led i, that is, $|\Delta N_{i\leftrightarrow j}|$. Node colours indicate ant social maturity, m.

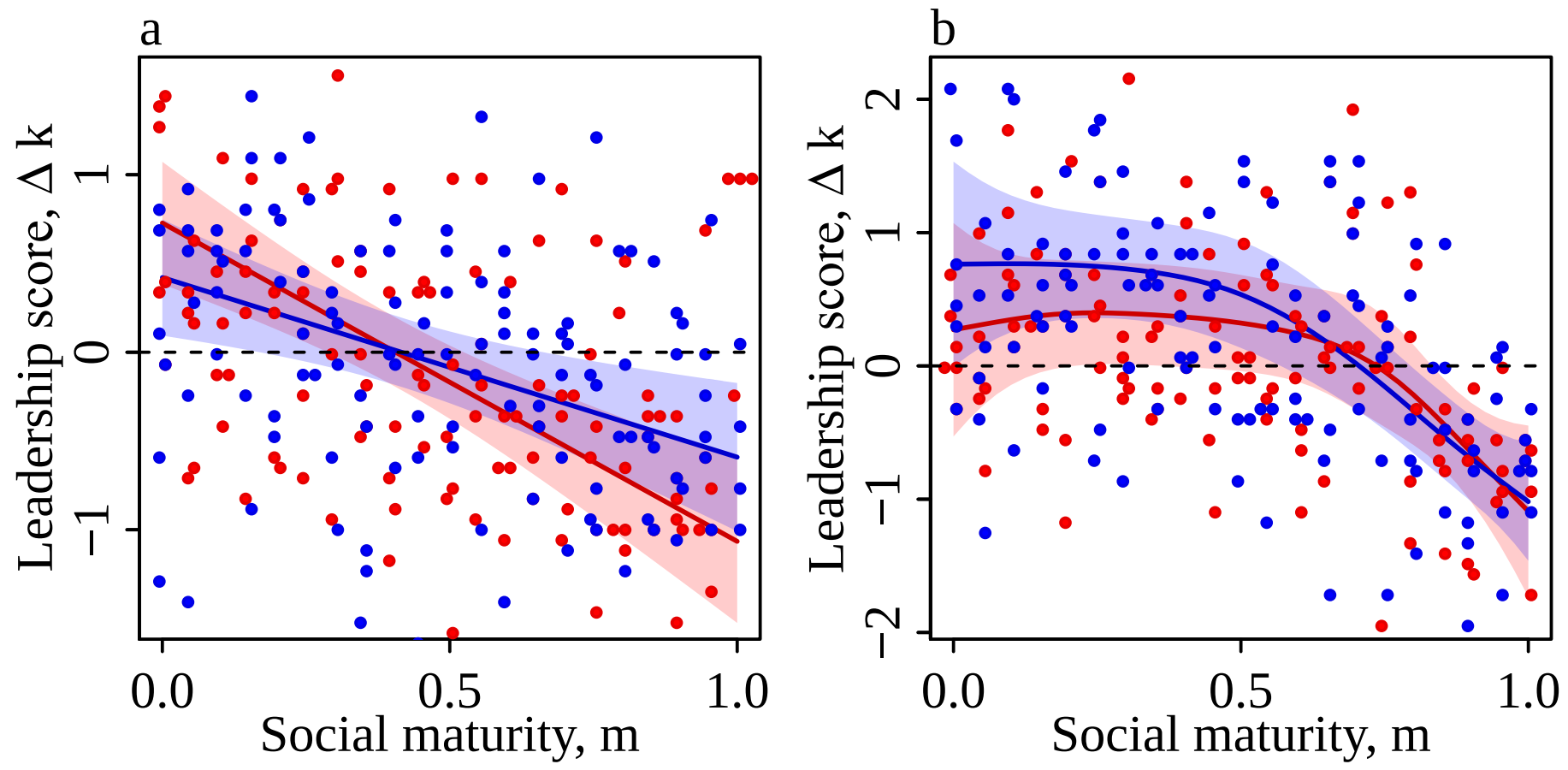

Figure S16: **Relationship between leadership score and social maturity for transmission networks generated from activity chains that remained confined within a single location.** Panel **(a)** shows the association between leadership score and social maturity for transmission networks constructed only from inner-to-inner activity chains, i.e., chains that were initiated in the inner location and did not spread to the outer location. Panel **(b)** shows the same for outer-to-outer chains. Points represent colony means. Red points - single-chamber nest. Blue points - double-chamber nest. Curved lines and shaded areas indicate the GAMM fits and 95% confidence intervals.

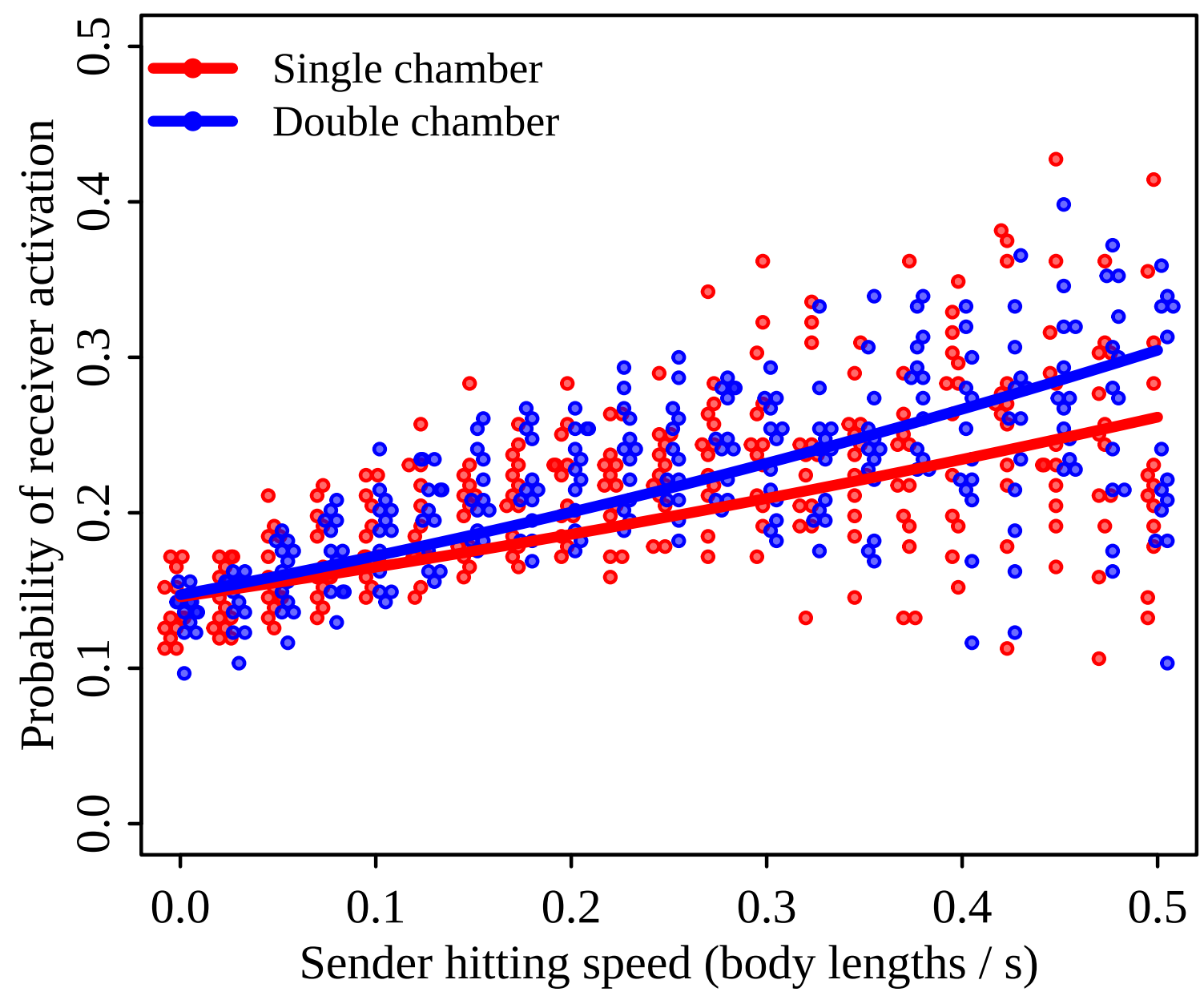

Figure S17: **Hitting speed predicts receiver activation.** Each point represents the mean for a single colony. Solid lines represent the fits from the GLMM described in the main text.
