# Supplementary material for: Modular nest structure influences activity cycle synchronisation in ant colonies
Source: Commun Biol. 2026 Jun 27;9:1317. doi: 10.1038/s42003-026-10560-x (PMC13649663; doi:10.1038/s42003-026-10560-x)
Supplement: Supplementary file 3 [file 42003_2026_10560_MOESM3_ESM.pdf]

Description of Additional Supplementary Files

File name: Supplementary Data

Description: The source data behind the graphs in the paper
