# Supplementary material for: Modular nest structure influences activity cycle synchronisation in ant colonies
Source: Commun Biol. 2026 Jun 27;9:1317. doi: 10.1038/s42003-026-10560-x (PMC13649663; doi:10.1038/s42003-026-10560-x)
Supplement: Supplementary file 4 [file 42003_2026_10560_MOESM5_ESM.pdf]

nature portfolio

Corresponding author(s): Nathalie Stroeymeyt, Thomas Owen Richardson

Last updated by author(s): May 29, 2026

Reporting Summary

Nature Portfolio wishes to improve the reproducibility of the work that we publish. This form provides structure for consistency and transparency in reporting. For further information on Nature Portfolio policies, see our Editorial Policies and the Editorial Policy Checklist.

Statistics

For all statistical analyses, confirm that the following items are present in the figure legend, table legend, main text, or Methods section.

n/a	Confirmed	
☐	☒	The exact sample size (n) for each experimental group/condition, given as a discrete number and unit of measurement
☐	☒	A statement on whether measurements were taken from distinct samples or whether the same sample was measured repeatedly
☐	☒	The statistical test(s) used AND whether they are one- or two-sided<br>*Only common tests should be described solely by name; describe more complex techniques in the Methods section.*
☐	☒	A description of all covariates tested
☐	☒	A description of any assumptions or corrections, such as tests of normality and adjustment for multiple comparisons
☐	☒	A full description of the statistical parameters including central tendency (e.g. means) or other basic estimates (e.g. regression coefficient) AND variation (e.g. standard deviation) or associated estimates of uncertainty (e.g. confidence intervals)
☐	☒	For null hypothesis testing, the test statistic (e.g. F, t, r) with confidence intervals, effect sizes, degrees of freedom and P value noted<br>*Give P values as exact values whenever suitable.*
☒	☐	For Bayesian analysis, information on the choice of priors and Markov chain Monte Carlo settings
☐	☒	For hierarchical and complex designs, identification of the appropriate level for tests and full reporting of outcomes
☒	☐	Estimates of effect sizes (e.g. Cohen's d, Pearson's r), indicating how they were calculated

Our web collection on statistics for biologists contains articles on many of the points above.

Software and code

Policy information about availability of computer code

Data collection	All data were collected using the protocols and software described in :<br>- Mersch, D. P., et.al. Tracking individuals shows spatial fidelity is a key regulator of ant social organization. Science 340, 1090 (2013).<br>- Stroeymeyt et al., Social network plasticity decreases disease transmission in a eusocial insect. Science 362(6417):941-945 (2018).<br>- Worker trajectories and physical contacts were extracted using freely available software; https://github.com/laurentkeller/anttrackingUNIL
Data analysis	The dataset and software pipeline used for the main analyses described in this study are available at https://doi.org/10.5281/zenodo.15020312.<br>Code for community detection using Facetnet is available at https://c4science.ch/source/facet_unil.

For manuscripts utilizing custom algorithms or software that are central to the research but not yet described in published literature, software must be made available to editors and reviewers. We strongly encourage code deposition in a community repository (e.g. GitHub). See the Nature Portfolio guidelines for submitting code & software for further information.

Data

Policy information about availability of data

All manuscripts must include a data availability statement. This statement should provide the following information, where applicable:

- Accession codes, unique identifiers, or web links for publicly available datasets
- A description of any restrictions on data availability
- For clinical datasets or third party data, please ensure that the statement adheres to our policy

The dataset and software pipeline used for the main analyses described in this study are available at https://doi.org/10.5281/zenodo.15020312.

Research involving human participants, their data, or biological material

Policy information about studies with human participants or human data. See also policy information about sex, gender (identity/presentation), and sexual orientation and race, ethnicity and racism.

Reporting on sex and gender	N/A
Reporting on race, ethnicity, or other socially relevant groupings	N/A
Population characteristics	N/A
Recruitment	N/A
Ethics oversight	N/A

Note that full information on the approval of the study protocol must also be provided in the manuscript.

Field-specific reporting

Please select the one below that is the best fit for your research. If you are not sure, read the appropriate sections before making your selection.

☒ Life sciences ☐ Behavioural & social sciences ☐ Ecological, evolutionary & environmental sciences

For a reference copy of the document with all sections, see nature.com/documents/nr-reporting-summary-flat.pdf

Life sciences study design

All studies must disclose on these points even when the disclosure is negative.

Sample size	We chose to use 13 colonies per experimental treatment as this represents a good practical compromise between sufficient replication at the colony level, and the time required to tag individuals that make up each colony. Previous studies using automated tracking data have used similar sample sizes (e.g., n=12 colonies per treatment in Stroeymeyt et al., Social network plasticity decreases disease transmission in a eusocial insect. Science 362(6417):941-945 (2018)).
Data exclusions	No data were excluded.
Replication	There were two levels of replication within our study: the level of the colony and the level of the individual. Biological replicates were carried out in parallel at the same time between the two treatments to avoid potential confounding effects associated with the date of experiments. Results have not been replicated by other labs at the time of submission.
Randomization	All colonies experienced both treatments successively, with half the colonies starting with one treatment and the other half starting with the other treatment. Allocation of colonies to starting treatments followed a pseudo-random procedure based on colony size, so demographics were similar across the two starting groups.
Blinding	All data collection and analyses were performed using automated procedures (automated tracking system; custom computer software) and were therefore not subject to any human biases.

Reporting for specific materials, systems and methods

We require information from authors about some types of materials, experimental systems and methods used in many studies. Here, indicate whether each material, system or method listed is relevant to your study. If you are not sure if a list item applies to your research, read the appropriate section before selecting a response.

Materials & experimental systems

n/a	Involved in the study
☒	☐ Antibodies
☒	☐ Eukaryotic cell lines
☒	☐ Palaeontology and archaeology
☐	☒ Animals and other organisms
☒	☐ Clinical data
☒	☐ Dual use research of concern
☒	☐ Plants

Methods

n/a	Involved in the study
☒	☐ ChIP-seq
☒	☐ Flow cytometry
☒	☐ MRI-based neuroimaging

Animals and other research organisms

Policy information about studies involving animals; ARRIVE guidelines recommended for reporting animal research, and Sex and Gender in Research

Laboratory animals	The study did not involve laboratory animals.
Wild animals	The study involved colonies of Leptothorax acervorum collected in Anzeindaz, Switzerland. Colonies were brought to the lab for experiments and kept there after the experiments ended.
Reporting on sex	N/A
Field-collected samples	Before and after the experiments, field-collected colonies were maintained under controlled laboratory conditions (14 L : 10D cycle, 25C, 55% RH) and housed in nests made of a cardboard perimeter sandwiched between two glass slides, delimiting a rectangular nest cavity of 36 x 48 x 1 mm with an entrance of 8 x 2 mm. They were kept in 155 x 135 x 50 mm plastic boxes with Fluon-coated walls to prevent the ants from escaping. They were provided with protein (Drosophila flies), diluted sugar water, and drinking water ad libitum.
Ethics oversight	The use of insects in research is not subject to any regulations or ethical restrictions in Switzerland.

Note that full information on the approval of the study protocol must also be provided in the manuscript.

Plants

Seed stocks	N/A
Novel plant genotypes	N/A
Authentication	N/A
